# Investigating Cu(II) Complexes for MRI: A Comprehensive Approach Using EPR, Relaxometry, and Computational Modeling

**DOI:** 10.1021/acs.inorgchem.5c05926

**Published:** 2026-03-03

**Authors:** Maria Chiara Pagliero, Marco Ricci, Raúl Alvarado, Carlos Platas-Iglesias, Enrico Salvadori, Valeria Lagostina, Mario Chiesa, Mauro Botta, Fabio Carniato

**Affiliations:** † Department of Chemistry, 9314 University of Turin, Via Giuria 9, 10125 Torino, Italy; ‡ Dipartimento di Scienze e Innovazione Tecnologica, 325870 Università del Piemonte Orientale, Viale Teresa Michel 11, 15121 Alessandria, Italy; § Centro Interdisciplinar de Química e Bioloxía (CICA) and Departamento de Química, Facultade de Ciencias, Universidade da Coruña, 15071 A Coruña, Galicia, Spain; ∥ Magnetic Resonance Platform (PRISMA-UPO), Università del Piemonte Orientale, 15121 Alessandria, Italy

## Abstract

The development of Gd-free MRI contrast agents requires a detailed understanding of the structural and electronic factors governing paramagnetic relaxation in first-row transition-metal complexes. In this work, we integrate EPR spectroscopy, Q-band ENDOR, variable-temperature ^17^O NMR, field-dependent ^1^H relaxometry, and DFT calculations to dissect the structure–relaxivity relationships of two prototypical Cu­(II) systems: [Cu­(TACN)]^2+^ and [Cu­(TREN)]^2+^. These complexes differ markedly in geometry, hydration state, and electronic ground state, offering a controlled platform to probe how the coordination environment modulates dipolar and scalar relaxation pathways. EPR and ENDOR measurements yield rotational correlation times and metal–proton hyperfine couplings in close agreement with theoretical predictions, enabling a quantitative description of water and proton exchange dynamics. ^1^H relaxometric analysis reveals distinct regimes. [Cu­(TACN)]^2+^ exhibits fast water exchange driven by a dynamic Jahn–Teller effect, whereas five-coordinate [Cu­(TREN)]^2+^ shows much slower exchange and a significant scalar contribution under basic conditions, where OH^–^ replaces inner-sphere water. Collectively, these results highlight the sensitivity of Cu­(II) relaxivity to subtle structural perturbations and demonstrate that targeted control of geometry and hydration can modulate inner-sphere and prototropic exchange pathways. The integrated methodology presented here provides a robust experimental–computational framework for the rational design of Cu­(II)-based MRI contrast agents.

## Graphical abstract

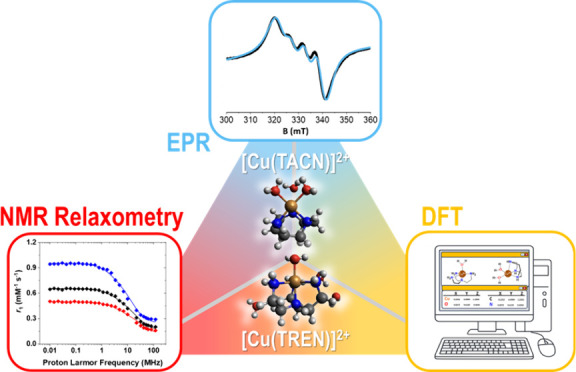

## Introduction

Magnetic resonance imaging (MRI) has rapidly become one of the most powerful and noninvasive techniques in clinical diagnostics. Its diagnostic precision and accuracy have been substantially enhanced by the development of highly efficient contrast agents (CAs). At present, the vast majority of clinically approved MRI contrast agents are based on Gd­(III) complexes, owing to their favorable electronic structure and high efficiency in enhancing proton relaxation rates.^1–4^ However, growing concerns regarding the long-term safety of gadolinium, particularly in patients with impaired renal function,^5,6^ have driven intensive research toward the development of alternative, Gd-free MRI contrast agents.^7–13^ Among these, Mn­(II) complexes have attracted considerable attention,^14–17^ although transition-metal-based systems more broadly remain comparatively underexplored.^18^

Although copper is one of the most abundant essential metals in biology and has long been studied for its therapeutic potential,^19^ Cu­(II) complexes have been largely overlooked as MRI contrast agents because of their presumed intrinsically low relaxivity.^20^ This perception has been reinforced by limited experimental data suggesting inefficient relaxation enhancement compared to Gd­(III). Recently, however, Peacock and co-workers^21^ reported the stabilization of an unusual Cu­(II) site within a synthetic protein scaffold, coordinated exclusively by oxygen donor atoms, that displayed remarkably high relaxivity, comparable to, and in some cases exceeding, that of clinical Gd­(III) agents. This striking finding challenges the long-standing assumption that Cu­(II) is unsuitable for MRI applications and highlights copper as a promising yet underexplored element for CA development.

Furthermore, the use of Cu­(II)-based MRI contrast agents could alleviate some of the concerns associated with the use of Gd­(III), although given the significant toxicity of copper,^22,23^ stable complexation of the metal ion is certainly required for potential clinical application. In addition, the redox potential for the reduction of Cu­(II) to Cu­(I) is a parameter that needs to be considered as well, as reduction of the metal ion by reducing agents present *in vivo* may provide a favorable path for complex dissociation.^24^ On the other hand, the redox chemistry of Cu­(II) complexes could be used to design contrast agents with response to the redox environment, as demonstrated for Mn­(II)/Mn­(III)^25,26^ and Fe­(II)/Fe­(III) complexes.^27^

The origin of the exceptional relaxivity mentioned above remains unclear. To address this fundamental question, it is essential to investigate a systematic library of small-molecule Cu­(II) complexes, in order to elucidate the structural and electronic factors that could enable a low-spin, low-magnetic-moment ion (*S* = 1/2) such as Cu­(II) to rival, or even approach, the performance of the high-spin Gd­(III) ion (*S* = 7/2) traditionally employed in MRI.

These considerations motivate a comprehensive exploration of the interplay between structure, electronic properties, and relaxivity in small-molecule Cu­(II) complexes. The Solomon-Bloembergen-Morgan (SBM) theory^28–30^ provides the theoretical framework linking relaxation enhancement to key structural and dynamic parameters, including metal-proton distances, electronic relaxation times, water-exchange rates, hyperfine interactions, and molecular reorientation dynamics.^31^ For Gd­(III) complexes, decades of research have yielded a coherent body of experimental and computational data, defining robust structure–function relationships and reliable design principles.^31,32^ In contrast, analogous systematic studies on Cu­(II) and other first-row transition-metal complexes remain scarce, and consequently, clear design rules are still lacking, despite the high sensitivity of relaxivity in these systems to subtle structural variations.

To address this gap, we recently introduced an integrated methodology combining electron paramagnetic resonance (EPR), ^1^H relaxometry, and computational chemistry to gain detailed insights into the structural, magnetic, and dynamic properties of paramagnetic complexes in aqueous solution.^33,34^ This comprehensive approach allows for the independent and experimental estimation of key parameters governing relaxivity, including metal-proton distances, the magnitude of metal–proton hyperfine interactions, and the rotational correlation times (τ_R_) of the complexes.^32^

In the present work, we apply this integrated methodology to two prototypical Cu­(II) complexes. These chelates were strategically selected to provide two Cu­(II) complexes with a comparable ligand environment and size, while stabilizing distinct coordination geometries and electronic ground states, enabling a focused analysis of how ground-state electronic structure impacts relaxivity. By systematically correlating the spectroscopic, relaxometric, and computational data for these distinct systems, our primary aim is to deepen the understanding of precisely how structural and magnetic factors influence relaxivity in complexes. Ultimately, this study contributes to the creation of a reliable structure-relaxivity data set, an essential prerequisite for establishing rational design principles for future Cu­(II)-based contrast agents.

## Experimental Section

### Materials

All chemicals were purchased from Sigma-Aldrich Co. and used without further purification.

### Synthesis of [Cu­(TACN)]^2+^ and [Cu­(TREN)]^2+^

50 mg of the ligand were first dissolved in 5 mL of Milli-Q water, and the pH of the solution was adjusted to 4.5 by the addition of 1.0 M NaOH. In parallel, 5 mL of an equimolar aqueous solution of CuCl_2_·2H_2_O was prepared. The copper solution was then added dropwise to the ligand under continuous stirring at room temperature. An immediate color change was observed, indicative of complex formation. The pH of the resulting mixture was subsequently adjusted to 4.0 to promote complete chelation of the Cu­(II) ions, and the solution was left stirring for 24 h at room temperature. The purification was performed by raising the pH to 9.0 with NaOH 1.0 M, to facilitate the precipitation of any residual free metal. After filtration of the solution, the pH was adjusted to 7.4 with HCl 1.0 M.

### Methods

Variable ^17^O NMR measurements were recorded on a Bruker AVANCE III 500 spectrometer equipped with a wide bore 11.7 T magnet. The aqueous solutions of the complexes were enriched to a final ^17^O (Cambridge Isotope) isotopic abundance of 2.0%.

The effective magnetic moment (μ_eff_) of the paramagnetic Cu­(II) complexes was determined using the BMS method.^35^ Solutions were prepared by mixing 188 μL of Cu­(II) chelate with a known concentration with 22 μL of a D_2_O solution containing 10% tert-butanol (used as a chemical shift reference) and 10 μL of Milli-Q water. The final mixture was transferred into a single 3 mm NMR tube. Proton NMR spectra were then acquired at 300 K, and the frequency shift between the tert-butanol resonance in the paramagnetic solution and in the absence of paramagnetic effects was measured.

1/*T*_1_^1^H nuclear magnetic relaxation dispersion (NMRD) profiles were measured on a Fast-Field Cycling (FFC) Stelar SmarTracer Relaxometer from 0.00024 to 0.25 T (0.01–10 MHz proton Larmor Frequencies). This relaxometer operates under computer control with a ± 1% uncertainty in 1/*T*_1_. The proton relaxation in the 20–120 MHz frequency range was investigated with a High Field Relaxometer (Stelar), equipped with a HTS-110 3T Metrology Cryogen-free Superconducting Magnet. The temperature during the measurements was controlled through a Stelar VTC-91 airflow heater equipped with a copper-constantan thermocouple (uncertainty of ± 0.1 °C). The real temperature inside the probe head was further monitored with a Fluke 52K/J digital thermometer (Fluke, Zürich, Switzerland). Data were collected using the standard inversion recovery sequence (16 experiments, 3 scans) with a typical 90° pulse width of 3.5 μs, and a reproducibility of the data within ± 0.5%. The diamagnetic contribution was measured by collecting ^1^H NMRD profiles of the solvent in absence of the paramagnetic complexes at different temperatures. Relaxivity values (*r*_1_, mM^–1^ s ^–1^) at different magnetic fields and temperatures were obtained by measuring the longitudinal relaxation rates of the sample solutions and subtracting the corresponding diamagnetic contribution depending on the measurement conditions, and by dividing this value for the mM concentration of Cu­(II).

The UV–vis spectra were obtained by using a Lambda 900 UV–Visible spectrometer (PerkinElmer, Waltham, Massachusetts, USA).

The copper content was determined with an Ametek Spectro Genesis EOP Inductively Coupled Plasma Atomic Emission Spectrometer (ICP-AES) (Kleve, Germany) equipped with a cross-flow nebulizer. The solutions were mineralized with HNO_3_ 70% at 373 K for 4 h and then diluted in 1 wt % HNO_3_ solutions before analysis.

*EPR Spectroscopy*. X-band (microwave frequency of 9.42 GHz) continuous-wave (CW)-EPR spectra were recorded at 298 and 77 K on a Bruker EMXmicro spectrometer. A modulation amplitude, modulation frequency and microwave power of 5 G, 100 kHz, 11 mW and 1.1 mW were used, respectively. Q-band (microwave frequency of 33.8 GHz) Pulse EPR experiments were obtained at 10 K with a Bruker ELEXYS 580 EPR spectrometer equipped with EN 5107D2 Bruker resonator and a cryogen-free variable temperature cryostat from Cryogenic Ltd. The magnetic field was measured with a Bruker ER035 M NMR gaussmeter. The electron-spin–echo (ESE) detected EPR spectra were recorded with the pulse sequence π/2-τ-π-τ-echo. The pulse lengths were *t*_π/2_ = 16 ns and *t*_π_ = 32 ns, a τ value of 200 ns and a shot repetition rate of 5 ms were used. Q-band electron nuclear double resonance (ENDOR) measurements were carried out at 10 K by employing the Davies pulse sequence (π-RF-π/2-τ-π-τ-echo), using a Bruker SpinJet-AWG. A Gaussian shaped pulse was used for the inversion pulse. For ^1^H nuclei, weakly coupled to the unpaired electron, the pulse length used were all set to *t*_π/2_ = 80 ns and *t*_π_= 160 ns. The RF pulse length was set to 14 μs and a resolution of 1000 points was adopted. Five mM solutions of [Cu­(TACN)]^2+^ and [Cu­(TREN)]^2+^ were prepared for both CW and pulse EPR experiments. Concerning low temperature measurements, 30% of glycerol was added to obtain good glasses.

All spectra were simulated and elaborated using the Easyspin package toolbox for MATLAB.^36^

*DFT Computations*. Density functional theory (DFT) calculations were performed using the ORCA package (release 6.0.0),^37–41^ which uses the SHARK^42^ integral package. In these calculations we used a mixed explicit/continuum approach that incorporated a few explicit water molecules, together with the SMD solvation model.^43,44^ Explicit water molecules were situated using the ORCA solvator tool with the aid of the semiempirical GFN2-xTB method.^45^ Geometry optimizations and subsequent frequency calculations on the [Cu­(TACN)­(H_2_O)_2_]^2+^·7H_2_O and [Cu­(TREN)­(H_2_O)]^2+^·7H_2_O systems were carried out with the TPSSh functional^46^ and the def2-TZVPP basis set.^47^ The resolution of identity and chain-of-spheres (RIJCOSX)^48–54^ approximation was used throughout in combination with the Def2/J^48^ auxiliary basis set. Atom-pairwise dispersion corrections were incorporated with the Becke-Johnson damping scheme (D3BJ).^55,56^

The Cu *A*- and *g*-tensors were calculated using relativistic calculations with the ZORA^57,58^ Hamiltonian and the zora-def2-TZVP basis set, which utilizes the exponents of the def2-TZVP^47^ basis set and was recontracted for ZORA calculations by D. A. Pantazis. The Cu *A*-tensors were calculated using the TPSS^46^ functional with the resolution of identity approximation, employing the AutoAux procedure^59^ to generate auxiliary basis sets. Conversely, the *g*-tensors were obtained with the double hybrid PBE0-DH functional^60^ with the RIJCOSX approximation in combination with AutoAux. Finally, ^1^H *A*-tensors were obtained at the TPSS/Def2-QZVPP level. The spin–orbit mean-field method (SOMF­(1X))^61,62^ was used to consider spin–orbit coupling contributions. All calculations of A- and g-tensors incorporated the SMD solvation model.^43,44^

*Molecular Dynamics*. All molecular dynamics simulations and subsequent analyses were performed using AMBER 20.13,^63^ following adapted protocols based on the methodology described by Lemkul.^64^ Quantum mechanical (QM) calculations were carried out with Gaussian 16,^65^ while molecular visualization and model preparation were performed using Avogadro^66^ and Chimera.^67^ The RESP charge derivation and parametrization of the Cu­(II) complexes through the MCPB.py framework were conducted using modules included in AMBERtools 22.2.^68^ The parametrization of the Cu­(II) complexes was based on the bonded model implemented in MCPB.py, ensuring compatibility with the GAFF force field.^69^ Missing bonded parameters involving the Cu­(II) ion—specifically bond and angle equilibrium values and force constants—were obtained from quantum mechanical calculations using the Seminario method.^70^ In this approach, the equilibrium bond lengths and angles were taken from the geometries optimized at the ωB97XD/def2-TZVPP level,^47,71^ while the force constants were extracted directly from the Hessian matrix of the complex, projected from Cartesian to internal coordinates. This ensures internal consistency and transferability between the quantum and molecular mechanical representations of the metal center. The RESP charge fitting was conducted independently from the Seminario step. Atomic charges were derived from the electrostatic potential (ESP) calculated at the same level of theory (ωB97XD/def2-TZVPP), using the Gaussian options *iop­(6/42 = 6)* and *iop­(6/33 = 2)*, as recommended in the GAFF development protocol to ensure coordinate-independent charges. The Cu­(II) van der Waals radius (1.391 Å) was assigned according to Bastanov et al.,^72^ optimized to reproduce the ion–oxygen distance in the OPC water model.^73^

Molecular dynamics simulations were performed within a cubic periodic box of water (30 Å per side), containing the Cu­(II) complex placed at least 10 Å away from the box boundaries. For the MCPB-based parametrization, the OPC water model was employed, setting the Lennard–Jones parameters of hydrogen atoms to zero. A total of 626 water molecules were included under both parametrization protocols, together with two chloride ions (Cl^–^) to neutralize the system. Initial energy minimization was performed using the steepest descent algorithm, followed by equilibration under NVT and NPT ensembles for 100 ps each with a 1 fs integration step. The production phase was conducted under NPT conditions using a 2 fs time step. Periodic boundary conditions were applied throughout, and long-range electrostatics were treated using the particle-mesh Ewald (PME) method.^74^ The Cu­(II) complex, solvent, and counterions were thermostated independently at 298.15 K. Lennard–Jones parameter optimization to reproduce the ion–oxygen distance (IOD) was achieved via 50 ns production simulations, excluding the initial 2 ns for equilibration. Radial distribution functions (RDFs) were computed using CPPTRAJ,^75^ the main AMBER analysis tool. The rotational correlation time (τ_R_) was derived from the autocorrelation function of the Cu–O vector, averaged over multiple 200 ps trajectory segments and fitted to a single-exponential decay.

## Results and Discussion

The combined use of EPR and ^1^H relaxometry represents a powerful approach for accurately determining the parameters that govern paramagnetic relaxation and for elucidating the underlying relaxation mechanisms of MRI probes in aqueous solution. The choice of [Cu­(TACN)]^2+^ and [Cu­(TREN)]^2+^ was strategically aimed at comparing two prototypical coordination environments: a rigid macrocycle versus a flexible ligand. This selection allows for a direct comparison between different coordination geometries (square pyramidal vs trigonal bipyramidal) and hydration states (*q* = 2 vs *q* = 1) (Figure 1). By using these well-defined scaffolds, our aim was to isolate the impact of the coordination symmetry and hydration state on the relaxation properties, by using a multitechnique strategy.

**Figure 1:**
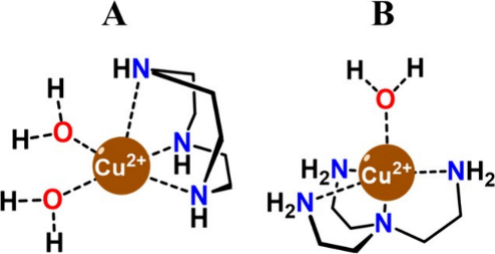
Chemical structures of (A) [Cu­(TACN)]^2+^ and (B) [Cu­(TREN)]^2+^.

Both complexes were synthesized by adding an equimolar aqueous solution of hydrated CuCl_2_ to a solution of the corresponding ligand at pH 4.0. After purification under basic conditions, the concentrations of the complexes in solution were determined by inductively coupled plasma optical emission spectroscopy (ICP–OES). The effective magnetic moments (μ_eff_) were calculated using Evans’ method,^35^ which relies on measuring the ^1^H NMR chemical shift of *tert*-butanol (*t*-BuOH) in solutions containing the paramagnetic complexes.^76^ [Cu­(TACN)­(H_2_O)_2_]^2+^ and [Cu­(TREN)­(H_2_O)]^2+^ complexes exhibit μ_eff_ values of 1.78 and 1.79, respectively, consistent with values reported for other Cu­(II) complexes.^77,78^

The [Cu­(TREN)]^2+^ complex is characterized by a good stability (logβ= 18.8),^79^ given by the chelating effect of tetradentate tripodal scaffold. Similarly, [Cu­(TACN)]^2+^ exhibits slightly lower stability (logβ= 15.4),^80^ given by the tridentate, macrocyclic nature of the ligand.

The [Cu­(TREN)­(H_2_O)]^2+^ complex adopts a compressed trigonal bipyramidal geometry, a distinctive feature of tripodal ligands reacting with Cu­(II). Crystallographic data on [Cu­(TREN)­(H_2_O)]^2+^-based complexes definitively shows this pentacoordinate (CN = 5) arrangement.^81^ Specifically, the tetradentate scaffold imposes a rigid coordination cage where the axial Cu–N (tertiary) bond is significantly shorter than the equatorial Cu–N (primary) bonds. Kinetic ^17^O NMR studies confirm that this specific geometry is maintained in aqueous solution,^82^ implying that the fifth coordination site, occupied by a single water molecule in the apical position, is subject to strong interactions.^83^ By contrast, the tridentate TACN macrocycle enforces a distinct coordination environment around the Cu­(II) ion. The X-ray structure of [Cu­(TACN)­Br_2_] complex^84^ reveals a distorted square pyramidal geometry, where the basal plane is defined by two nitrogen atoms of the ring and the two bromide ligands, while the third nitrogen occupies the apical position. Notably, the apical Cu–N bond is significantly elongated compared to the equatorial ones. Based on this structural evidence, it is reasonable to assume that the two equatorial coordination sites, occupied by bromide ions in the solid state, are readily accessible to solvent molecules in aqueous media. This supports the formation of a bis-aqua [Cu­(TACN)­(H_2_O)_2_]^2+^ (*q* = 2). Recent investigations on similar macrocyclic derivatives further corroborate this coordination model, supporting this pentacoordinate geometry fashion in aqueous solution.^85^

In aqueous solution, paramagnetic complexes interact with both coordinated (inner-sphere, IS) and noncoordinated (outer-sphere, OS) water molecules (eq 1). For hydrated complexes, the IS contribution typically dominates the enhancement of the longitudinal relaxation rate (*R*_1_) of water protons.^31^ This relaxation depends on the number of coordinated water molecules (*q*), the concentration of the paramagnetic probe (*c*), the residence lifetime of the inner-sphere water (τ_M_) and the longitudinal relaxation time of the bound water protons (*T*_1M_) (eq 2).

$$R_{1}=R_{1}^{\mathrm{IS}}+R_{1}^{OS}$$ 1

$$R_{1}^{\mathrm{IS}}=\frac{cq}{55.56}\frac{1}{T_{1M}+\tau_{M}}$$ 2

The Solomon–Bloembergen–Morgan (SBM) model remains the most widely used theoretical framework for describing paramagnetic relaxation mechanisms.^28–30^ It accounts for nuclear spin relaxation as the sum of dipole–dipole (DD), scalar (SC), and Curie (Cu) contributions.^86^ In *S* = 1/2 systems, however, the Curie term is typically negligible, as it is much smaller than the dipolar contribution.^31^ The dipolar mechanism, particularly relevant at high magnetic fields (B_0_ > 0.5 T), is influenced by several dynamic parameters, including the lifetime of the inner-sphere water molecule (τ_M_), the rotational correlation time of the complex (τ_R_) in solution, and the electronic relaxation time (τ_S_), all of which are closely related to the chemical and structural properties of the paramagnetic probe.^32^

Key parameters governing proton relaxivity, such as the rotational correlation time, the metal–proton distance, and spin density delocalization, can be directly determined using EPR and related hyperfine spectroscopic techniques such as ENDOR, as discussed below.

### EPR and ENDOR Characterization of [Cu­(TACN)]^2+^ and [Cu­(TREN)]^2+^

EPR spectra of Cu­(II) are characterized by an anisotropic ***g*** matrix and a hyperfine tensor (^**Cu**^ ***A***) due to coupling of the electronic spin *S* = 1/2 with the nuclear spin *I* = 3/2 of both copper isotopes (^63^Cu and ^65^Cu, with natural abundances 69.17% and 30.83% respectively). The hyperfine interactions with the nitrogen ligands are not resolved in the CW-EPR spectra and were therefore investigated using ELDOR-detected NMR experiments (Figures S3 and S4). Owing to the small magnitude of the ^14^N hyperfine couplings, which remain unresolved in CW-EPR, the spectral simulations were performed by considering only the ^63,65^Cu hyperfine interaction, as described by the following spin Hamiltonian:

$$H=\beta_{e}B_{0}gS+SAI$$ 3

The EPR spectrum of the [Cu­(TACN)]^2+^ complex, recorded in water frozen solution (77 K) at pH 4.5 (Figure 2c), reveals features characteristic of Cu­(II) (3d^9^) with square pyramidal geometry, with *g*_||_ > *g*_⊥_ > *g*_e_ as well as a hyperfine tensor with *A*_*x*_ = *A*_*y*_ ≪ *A*_*z*_ (Table 1), diagnostic of a semioccupied molecular orbital (SOMO) predominantly Cu *d*x^2^-y^2^ in character. The corresponding dynamically averaged, room temperature, spectrum is reported in Figure 2d along with the corresponding computer simulation. Under these circumstances, the anisotropic *g*- and *A*-tensors are averaged out depending on the rotational correlation time (τ_R_) of the complex. This results in a 4-line spectrum centered at the average < *g* > factor and line separation reflecting the Cu isotropic hyperfine component (*a*_iso_). Using the rigid limit spin-Hamiltonian parameters reported in Table 1, τ_R_ was determined through the simulation of the spectrum leaving τ_R_ as a single adjustable parameter. In this way a τ_R_ of 7.6 ps was estimated at 298 K.

**Figure 2:**
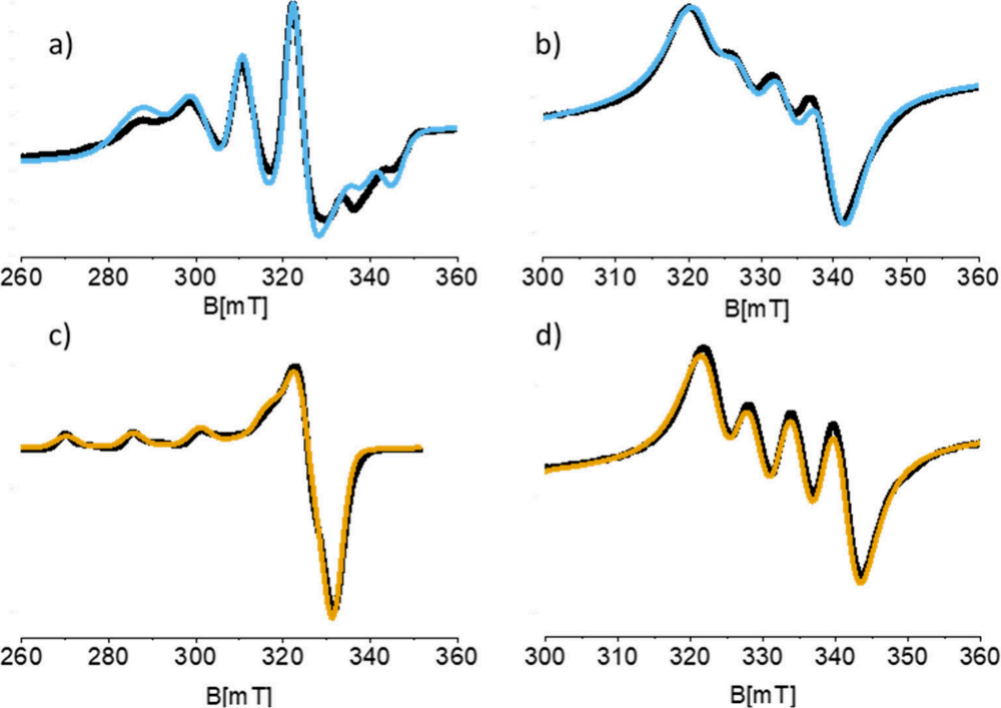
X-band CW-EPR spectra of (a) a water frozen solution (77 K) of [Cu­(TREN)]^2+^, (b) the same solution at 298 K, (c) a water frozen solution (77 K) of [Cu­(TACN)]^2+^, and (d) the same solution at 298 K. Experimental spectra are colored black, and simulated spectra are colored light blue and yellow. The parameters used for the simulations are listed in Table 1.

**Table 1:** Values of *g* and ^Cu^ *A* Used for the Simulation of the CW-X Band EPR Spectra Shown in Figure 2 ^a^

		⟨*g*⟩_av_	*a*_iso_	*g*_*x*_	*g*_*y*_	*g*_*z*_	^Cu^ *A*_*x*_	^Cu^ *A*_*y*_	^Cu^ *A*_*z*_	τ_R_
[Cu(TACN)]^2+^	Exp.	2.14	181	2.058 ± 0.004	2.058 ± 0.004	2.288 ± 0.008	30 ± 5	30 ± 5	–482 ± 20	7.6
	DFT	2.131	–145	2.067	2.077	2.249	33	60	–529	5.8^b^
[Cu(TREN)]^2+^	Exp.	2.137	–166	2.191 ± 0.008	2.207 ± 0.006	2.005 ± 0.005	–346 ± 8	–332 ± 8	180 ± 10	13
	DFT	2.135	–100	2.182	2.218	2.005	–335	–222	256	16.6^b^

a The ^N^ *A* values extracted from EDNMR experiments are listed in Table S1. All hyperfine values are given in units of MHz, while *τ*_R_ is in ps. The *g*- and *A*-tensors were calculated at the PBE0-DH/ZORA-Def2TZVP and TPSS/ZORA-Def2TZVP levels, respectively. The absolute sign of the experimental hyperfine tensor components has been taken in accord with the DFT results.

b Values calculated with classical MD simulations.

The corresponding experiments performed for the [Cu­(TREN)]^2+^ complex at pH 7 provide spin Hamiltonian parameters (Table 1) typical for trigonal bipyramidal copper complexes^87–90^ characterized by a *g* tensor structure with g_*x*_, g_*y*_ > g_*z*_ ≈ 2 and hyperfine structure with *A*_*x*_ ≈ *A*_*y*_ > *A*_*z*_, diagnostic of a SOMO with dominant contribution of the Cu *d*z^2^ orbital, in line with the DFT results discussed below. The small rhombicity of the *g* tensor obtained from spectral simulations indicates a slight deviation from the ideal *D*_3h_ symmetry expected for a trigonal bipyramidal structure. The corresponding room-temperature EPR spectrum is shown in Figure 2b, together with its simulated fit, yielding a rotational correlation time τ_R_ ≈ 13 ps. This value is consistent with those reported for Cu­(II) complexes of comparable size in aqueous solution. Table 1 compares the experimentally derived parameters with those obtained from DFT calculations for the two structures (vide infra).

Key parameters determining the proton relaxivity of coordinated water molecules are the scalar hyperfine coupling constant (*a*_iso_) and the metal-proton distance. Both can be directly measured using hyperfine spectroscopic techniques. In this study, Davies ENDOR experiments at Q-band frequency were employed to determine the relevant proton hyperfine couplings. Orientationally selective ENDOR spectra were recorded at different magnetic field positions, as shown in Figure 3 for both [Cu­(TACN)]^2+^ (Figure 3a-d) and [Cu­(TREN)]^2+^ (Figure 3e-h).

**Figure 3:**
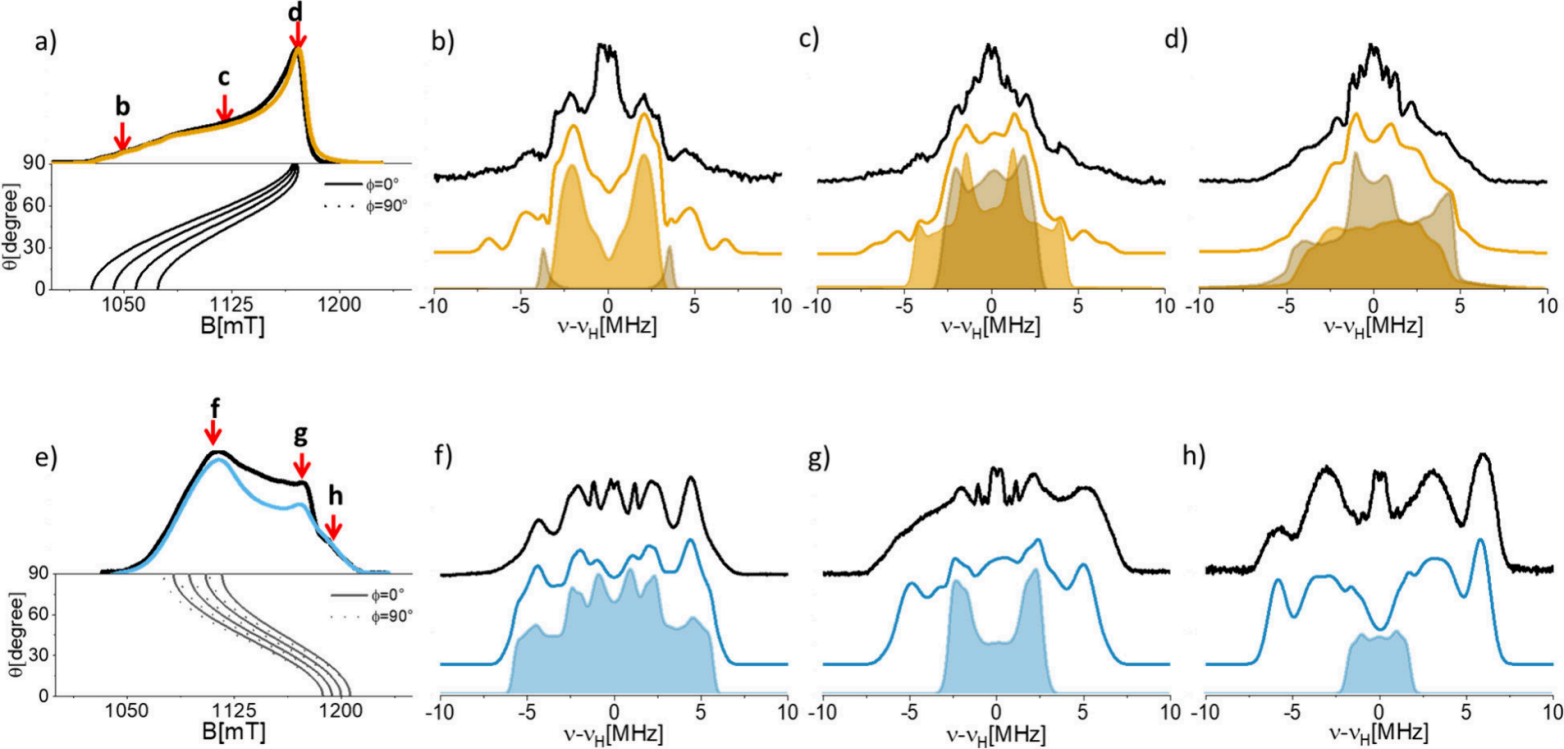
(a) Q-band ESE detected EPR spectrum of a [Cu­(TACN)]^2+^ water frozen solution. The simulation (yellow) was obtained using the SH parameters determined from the X-band CW spectrum (Table 1). The angular dependency of the resonance absorption is shown below the spectrum. The arrows indicate the magnetic field positions at which the ^1^H Davies ENDOR spectra (b–d) were recorded. (e) Q-band ESE detected EPR spectrum of a [Cu­(TREN)]^2+^ water frozen solution and (f–h) ^1^H Q-band Davies ENDOR spectra recorded at the magnetic field positions indicated in panel e. All experiments were performed at 10 K.

All samples show complex ^1^H ENDOR patterns, characterized by doublets centered at the proton nuclear Larmor frequency (ν_n_(^1^H) ∼ 51 MHz at 1200 mT) and split by the effective, orientation-selective hyperfine coupling *A*, as expected for weakly coupled nuclei (|*A*| < 2|ν_n_|).

The complex ^1^H ENDOR pattern arises from the superposition of signals originating from both the exchangeable protons of coordinated water molecules and the exchangeable and nonexchangeable protons of the ligand scaffold. The exchangeable scaffold protons are associated with amino (−NH) groups, whereas the nonexchangeable ones are bonded to carbon atoms. In this study, our focus is on the magnetic interactions involving water protons, as these directly govern relaxivity. However, because the (−NH) protons of the ligand framework undergo rapid H/D exchange, deuteration cannot be employed to selectively isolate the water-derived signals. Moreover, accurate ENDOR spectral simulations require at least six independent parameters per proton, three principal components of the hyperfine tensor and three Euler angles defining the orientation between the ***A*** and ***g*** tensors. In the absence of a reliable structural model, choosing these parameters lacks a sound physical basis. To address this, we performed DFT calculations on energy-minimized structures and used the computed hyperfine parameters as guidance for simulating the experimental ^1^H ENDOR spectra. The geometry and optimized structures are shown in Figure 4 and the full set of calculated spin-Hamiltonian parameters are listed in the Supporting Information.

**Figure 4:**
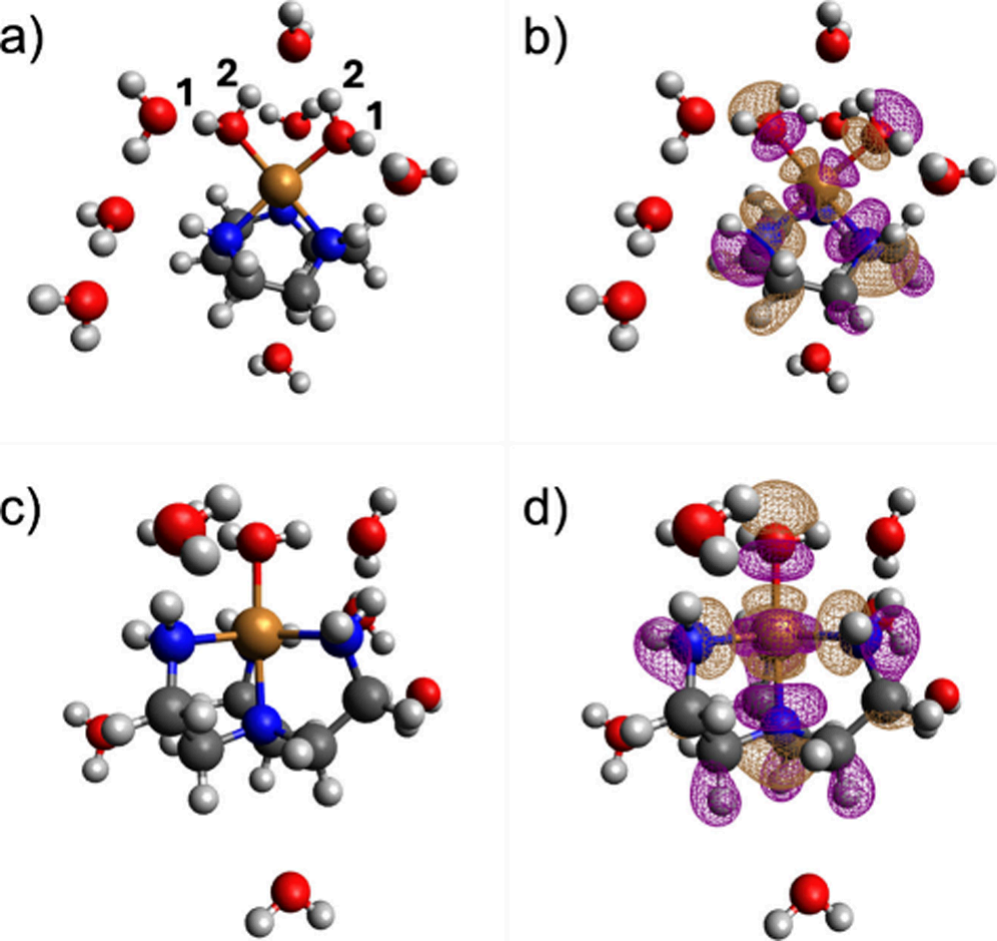
Geometries of (a) [Cu­(TACN)­(H_2_O)_2_]^2+^·7H_2_O and (c) [Cu­(TREN)­(H_2_O)]^2+^·7H_2_O obtained from geometry optimizations at the TPSSh/Def2-TZVP level and isodensity plots (0.02 a. u.) of the SOMOs of the quasi-restricted orbitals of (b) [Cu­(TACN)­(H_2_O)_2_]^2+^·7H_2_O and (d) [Cu­(TREN)­(H_2_O)]^2+^·7H_2_O. The numbers identify the water protons providing hyperfine couplings in the Q-band ENDOR spectra of [Cu­(TACN)­(H_2_O)_2_]^2+^.

The energy-minimized structures were evaluated by comparing their computed EPR parameters with those obtained from spectral simulations (Table 1), namely the *g*-values and the hyperfine couplings to the Cu nucleus. A good agreement between experimental and computed values is obtained for [Cu­(TACN)]^2+^. For the [Cu­(TREN)]^2+^ complex, the calculated anisotropic hyperfine components *A*_y_ and *A*_z_ deviate more from experiment than *A*_x_. This behavior reflects the electronic and geometric lability of this system, which lies close to the borderline between square-pyramidal and trigonal-bipyramidal coordination. In such cases, small structural variations lead to significant mixing between the *d*x^2^-y^2^ and *d*z^2^ orbitals, to which *A*_*y*_ and *A*_*z*_ are particularly sensitive. In addition, the experimental EPR parameters represent an average over a distribution of conformations and low-lying vibronic states in frozen solution, whereas the DFT calculations refer to a single optimized structure in the electronic ground state. Despite these limitations, the agreement obtained for the g-tensor and for *A*_*x*_ indicates that the adopted structural model captures the essential features of the electronic structure of the Cu­(II) center and lends confidence to the energy-minimized structures shown in Figure 4.

The spin-Hamiltonian parameters derived from the DFT-optimized models for the different proton types (water, amino, and CH) were subsequently used as input for the ENDOR spectral simulations. In these simulations, the Euler angles predicted by DFT were kept fixed, while the hyperfine tensor components were allowed to vary to best reproduce the position and relative intensity of the experimental ENDOR lines. The resulting values can therefore be considered as boundary estimates for the magnitude of the hyperfine tensors. The simulated spectra are shown in Figure 3, and the corresponding parameters are compared with the DFT-predicted values in Table 2. Only minor adjustments were required for the water and amino protons, whereas the isotropic hyperfine couplings of the carbon-bound protons were systematically overestimated by DFT. This discrepancy likely arises from small deviations in the DFT-predicted distances and orientations of the −CH groups relative to the experimental structure.

**Table 2:** Spin-Hamiltonian Parameters Used in the Simulation of the Q-Band ENDOR Spectra Shown in Figure 3 ^a^

			^H^ *A_x_*	^H^ *A*_*y*_	^H^ *A*_*z*_	α	β	γ	*a*_iso_	*r*_Cu–H_
[Cu(TACN)]^2+^	^1^H_(H2O)1_	SIM	–2.80 ± 0.3	8.75 ± 0.8	–8.5 ± 0.8	47.3	47.3	–18.4	–0.85	2.59
		DFT	–2.89	7.47	–8.32				–1.2	2.54
	^1^H_(H2O)2_	SIM	9.5 ± 1	–1.95 ± 0.2	–7.5 ± 0.5	–162.5	9.8	–154.5	0.02	2.61
		DFT	9.03	–0.95	–7.23				0.3	2.59
[Cu(TREN)]^2+^	H_2_O	SIM	–5.2 ± 0.3	–11.8 ± 1	5.7 ± 0.4	4.3	44.7	–1.3	–3.8	2.61
		DFT	–5.19	–12.3	6.69				–3.6	2.48

a Hyperfine couplings are given in MHz, and the Euler angles in deg.

For the [Cu­(TACN)]^2+^ complex, two water molecules are bound in the first coordination sphere, yielding two pairs of equivalent protons (labeled in Figure 4). Likewise, two of the amino protons are equivalent while the third is distinct. In the simulations presented in Figure 3, only the contribution of the most strongly coupled protons is included. Weakly coupled protons, responsible for the transitions in the central region of the spectrum (±2 MHz), were neglected. Such weakly coupled protons can be associated with both second shell water molecules and C–H protons from the ligand scaffold, as emerges from DFT calculations. Despite this limitation, this approach provides a fair description of the experimental spectrum and allows unambiguous identification of the water protons, highlighted as shaded areas in the figure.

A similar strategy was applied to the [Cu­(TREN)]^2+^ complex, where a single water molecule contributes with one pair of equivalent protons. The simulation of the individual components for both spectra is shown in Figures S1 and S2 and Table S2 and S3.

### Relaxometric Characterization of [Cu­(TACN)]^2+^ and [Cu­(TREN)]^2+^

The pH dependence of the relaxivity (*r*_1_) was initially investigated for the [Cu­(TACN)]^2+^ complex, as this analysis provides valuable insight into its stability in aqueous solution and reveals possible geometric or coordination changes induced by acidic or basic conditions (Figure 5A). A distinctive pH-dependent trend in relaxivity was observed: *r*_1_ increases at pH values below 3.0, reaches a plateau between pH 3.0 and 6.0, and then decreases before stabilizing again at basic pH values above 8.0, with a slight increase at very alkaline pH (>10.5). This behavior suggests pH-driven modifications in the structure and hydration state of the complex. Supporting evidence comes from UV–Visible spectra recorded over the pH range 2.0–12.0 (Figure 5 B, C). The combined analysis of relaxometric and spectroscopic data allows the complex’s behavior to be interpreted in terms of three distinct pH regions, each corresponding to specific structural or coordination features.

**Figure 5:**
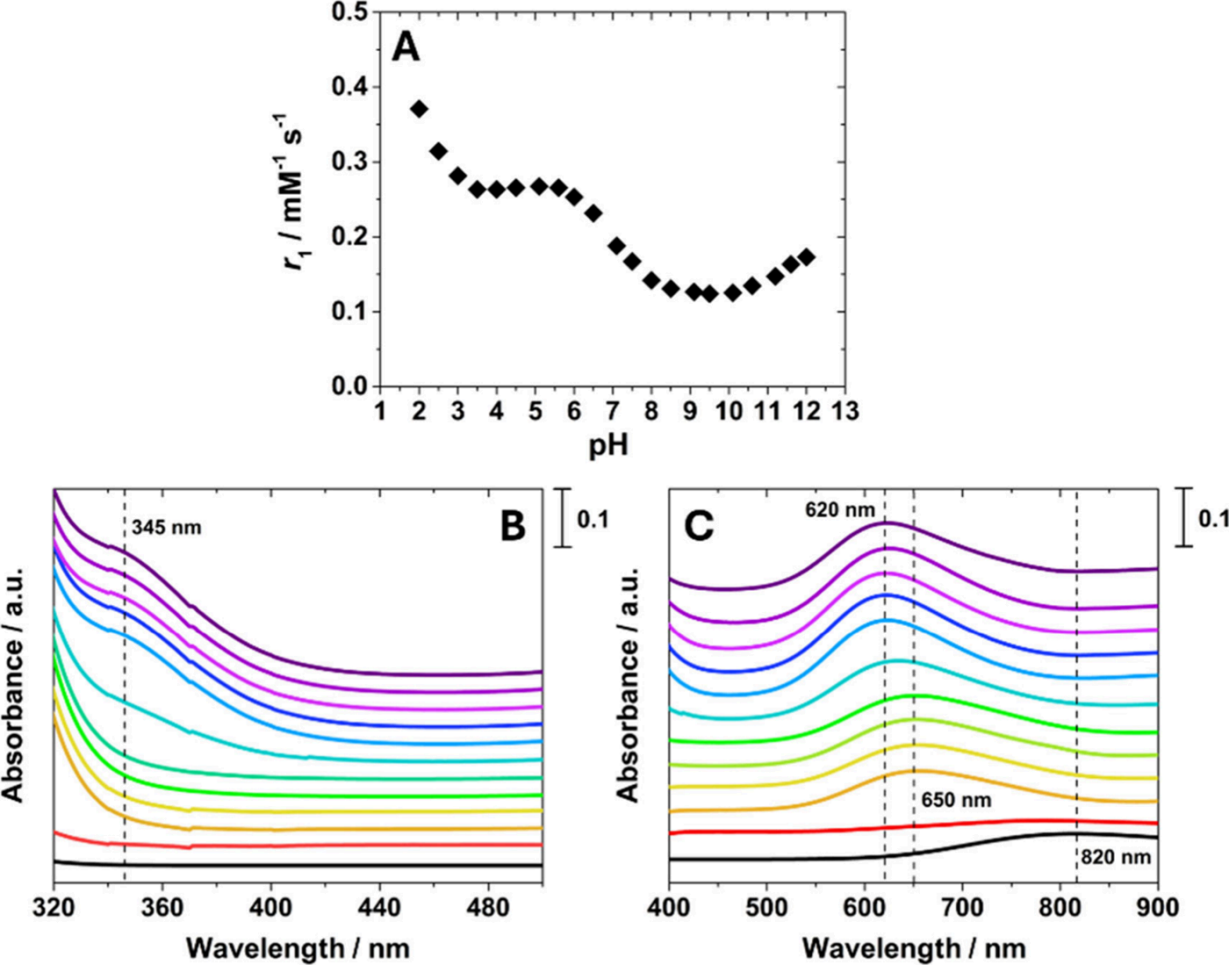
(A) pH dependence of *r*_1_ for [Cu­(TACN)]^2+^, recorded at 32 MHz and 298 K ([Cu­(II)] = 31.3 mM). (B and C) UV–visible spectra of [Cu­(TACN)]^2+^ recorded as a function of pH from 2.0 (red) to 12.0 (violet), with stepwise increases of one unit ([Cu^2+^] = 1.3 mM). The spectrum of [Cu­(H_2_O)_6_]^2+^ is reported for comparison (black line) ([Cu­(II)] = 4.0 mM). For the sake of clarity, the UV–visible spectra are vertically shifted to better illustrate differences in the curves.

At pH values around 2.0, decomplexation of the metal ion occurs, leading to an increase in *r*_1_ due to the higher hydration of the free Cu­(II) aquo ion compared with the coordinated [Cu­(TACN)]^2+^ complex. In this pH range, the UV–Vis spectrum no longer shows the characteristic *d–d* transition of the complex near 650 nm, but instead exhibits a broad band centered at 820 nm with lower molar absorptivity (ε) values. This spectral feature is typical of the hydrated Cu­(II) ion, further supporting the occurrence of decomplexation. Between pH 2.0 and 3.0, *r*_1_ decreases as copper ions progressively coordinate with the TACN ligand. From pH 3.0 to 6.0, [Cu­(TACN)]^2+^ displays a constant *r*_1_ value of 0.26 mM^–1^ s^–1^ at 32 MHz and 298 K, indicating that the hydration state of the complex remains unchanged across this range. The corresponding UV–Vis spectra confirm the presence of the characteristic *d–d* transitions of [Cu­(TACN)]^2+^.^91^

At pH values above 6.0, *r*_1_ decreases again, consistent with the gradual deprotonation of water molecules coordinated to the metal center. Beyond pH 8.0, a new plateau is observed, suggesting the predominant formation of the [Cu­(TACN)­(OH)]^+^ species. The UV–vis spectra in this region display a red shift of the absorption maximum to 620 nm and the appearance of a shoulder at 345 nm, both consistent with the presence of coordinated hydroxide ligands and in agreement with previous reports on Cu­(II)-TACN and its derivatives.^85,91^ Finally, the slight increase in *r*_1_ observed under highly basic conditions (pH > 10.0) can be attributed to an OH^–^-catalyzed proton exchange process, likely involving the amine protons, which enhances the relaxivity of the complex.^92^

A similar strategy was applied to characterize the [Cu­(TREN)]^2+^ complex. The *r*_1_ values as a function of pH reveal a behavior comparable to that observed for [Cu­(TACN)]^2+^. Below pH 4.5, a marked increase in *r*_1_ is observed, attributable to the gradual decomplexation of [Cu­(TREN)]^2+^ and the formation of the Cu­(II) aqua ion (Figure 6A). Between pH 4.5 and 8.0, a broad plateau of relaxivity around 0.2 mM^–1^s^–1^ indicates good stability of the complex within this range. At higher pH values, a gradual decrease in *r*_1_ occurs, consistent with deprotonation of the water molecule coordinated to the metal center. The UV–Vis absorption spectra of [Cu­(TREN)]^2+^ as a function of pH support this interpretation and provide further insights into the coordination properties of the complex (Figure 6B).

**Figure 6:**
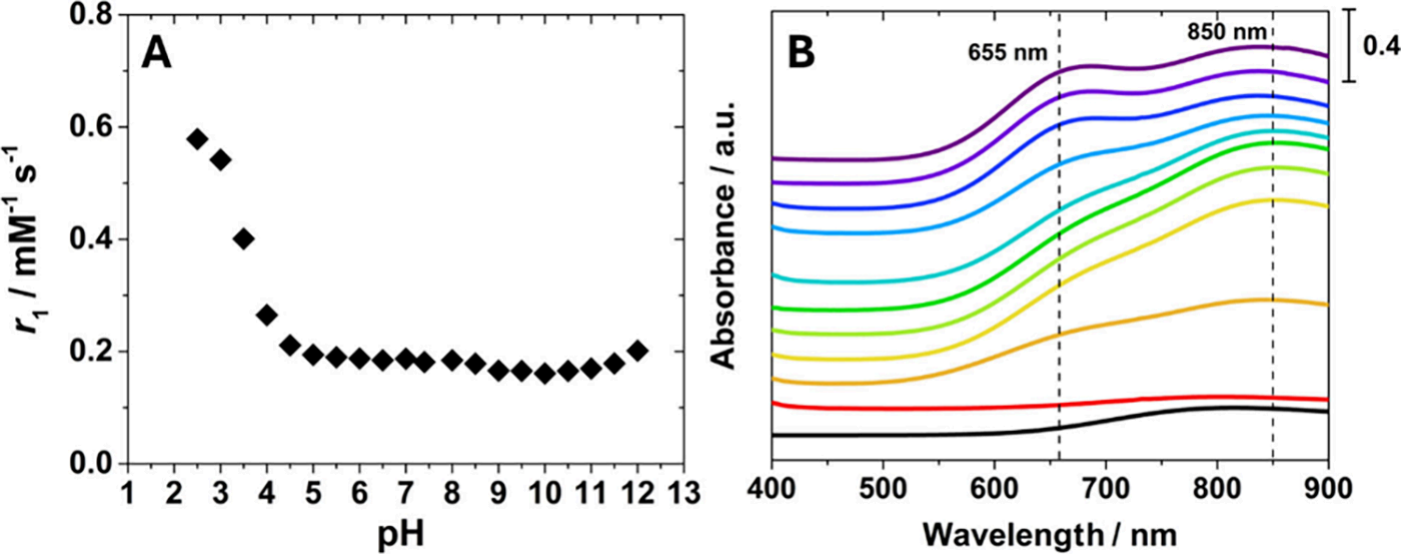
(A) pH dependence of *r*_1_ for [Cu­(TREN)]^2+^, recorded at 32 MHz and 298 K ([Cu­(II)] = 22.5 mM). (B) Visible spectra of [Cu­(TREN)]^2+^ recorded as a function of pH from 3.0 (red) to 12.0 (violet), with stepwise increases of one unit ([Cu­(II)] = 3.0 mM). The visible spectrum of [Cu­(H_2_O)_6_]^2+^ is reported for comparison (black line) ([Cu­(II)] = 11.0 mM). For the sake of clarity, the UV–visible spectra are vertically shifted to better illustrate differences in the curves.

Between pH 2.0 and 5.0, the spectra show the progressive complexation of the Cu­(II) aqua ion by the ligand. Near neutral pH, the spectra display the characteristic absorption pattern of trigonal–bipyramidal Cu­(II) complexes typical of tripodal ligands such as TREN,^83^ with a main band centered at 850 nm and a shoulder at 655 nm (Figure 6B). As documented in the literature, pentacoordinate Cu­(II) complexes with coordination geometries intermediate between trigonal-bipyramidal and square-pyramidal show two *d-d* transitions of comparable intensity between 650 and 1000 nm.^93^ Notably, as pH increases, the absorbance ratio of the 850 and 655 nm bands changes (Figure 6B), most likely due to deprotonation of the inner-sphere water molecule and subsequent coordination of OH^–^ to the metal ion, as also observed for [Cu­(TACN)]^2+^.

The [Cu­(TACN)]^2+^ and [Cu­(TREN)]^2+^ complexes were further characterized by relaxometry in their hydrated forms (pH 4.5 and 7.0, respectively). The dependence of *r*_1_ on the applied magnetic field and temperature, known as the Nuclear Magnetic Relaxation Dispersion (NMRD) profile, provides insight into the molecular dynamics governing paramagnetic relaxation. Fitting these profiles using the standard Solomon-Bloembergen-Morgan (SBM) model^28–30^ allows extraction of key molecular parameters. The ^1^H NMRD profiles of [Cu­(TACN)]^2+^ and [Cu­(TREN)]^2+^ are shown in Figures 7A and 8A, respectively. Given the pronounced pH-dependent variations in relaxivity observed for [Cu­(TREN)]^2+^, an additional relaxometric characterization was performed at pH 12.0 to assess the influence of hydroxo-species formation on its relaxometric properties. Under these conditions, both ^1^H NMRD and ^17^O variable-temperature (VT) NMR profiles were acquired, revealing marked differences compared to the measurements at neutral pH (Figure 8).

**Figure 7:**
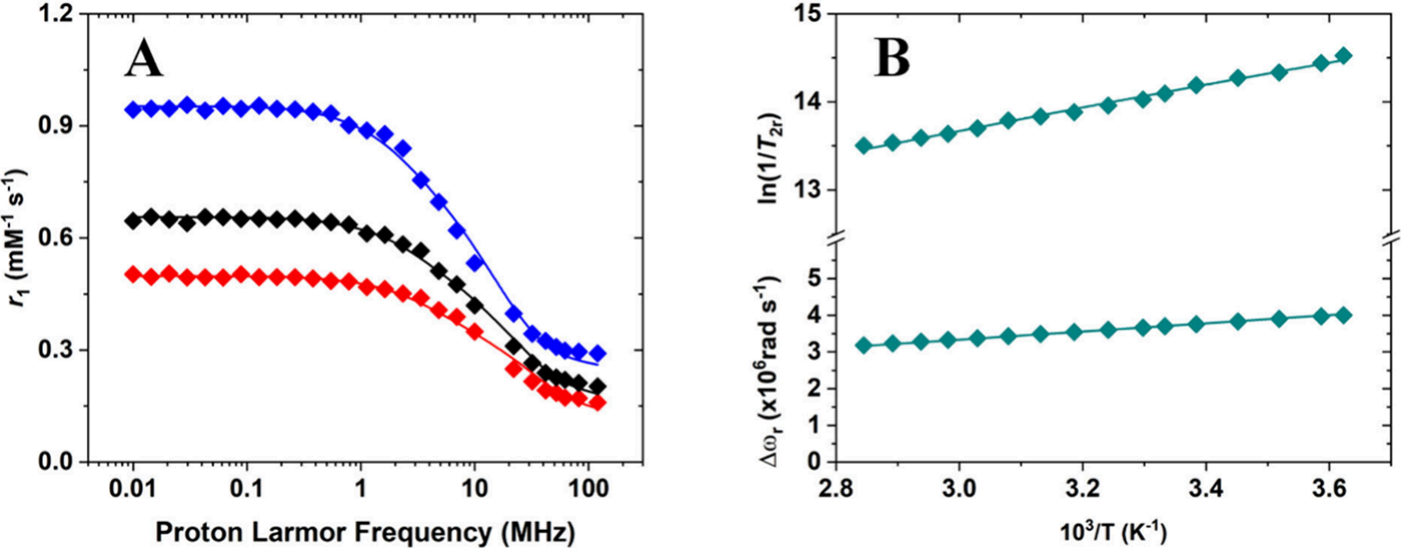
(A) ^1^H NMRD profiles of [Cu­(TACN)]^2+^ at (blue) 283 K, (black) 298 K, and (red) 310 K recorded at pH 4.5 and 31.3 mM Cu­(II). (B) ^17^O reduced transverse relaxation rates and chemical shifts measured at 11.7 T as a function of temperature for [Cu­(TACN)]^2+^ ([Cu­(II)] = 26.7 mM).

**Figure 8:**
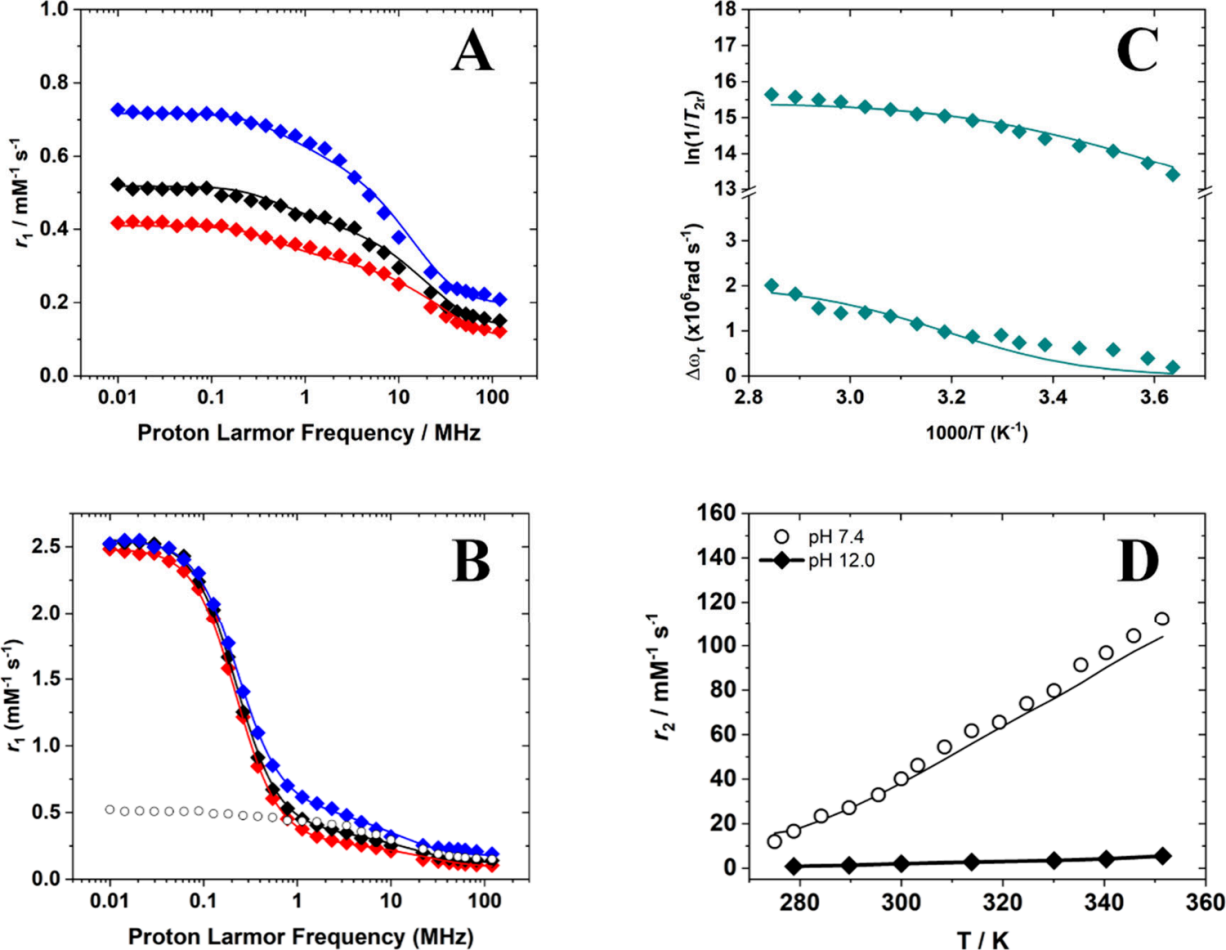
(A) ^1^H NMRD profiles of [Cu­(TREN)]^2+^ at (blue) 283 K, (black) 298 K, and (red) 310 K recorded at pH 7.4 and 22.5 mM Cu­(II). (B) ^1^H NMRD profiles of [Cu­(TREN)]^2+^ at (blue) 283 K, (black) 298 K, and (red) 310 K recorded at pH 12.0 and 22.5 mM Cu­(II). The ^1^H NMRD of the complex at neutral pH and 298 K is reported for comparison (○). (C) ^17^O reduced transverse relaxation rates and chemical shift measured at 11.7 T as a function of temperature for [Cu­(TREN)]^2+^ (26.7 mM Cu­(II), pH 7.4). (D) Comparison of ^17^O *r*_2_ values recorded for [Cu­(TREN)]^2+^ at (◆) pH 7.4 and (○) pH 12.0.

The ^1^H NMRD profiles recorded for the hydrated [Cu­(TACN)]^2+^ and [Cu­(TREN)]^2+^ complexes display the characteristic features of low-molecular-weight paramagnetic probes: a plateau at low magnetic fields (0.01–0.1 MHz), a dispersion centered around 4–5 MHz, and a second plateau at higher fields (>30–40 MHz). The presence of a single main dispersion indicates that the observed *r*_1_ values are predominantly governed by dipolar relaxation mechanisms. However, in the case of [Cu­(TREN)]^2+^, a faint additional dispersion in the 0.01–0.1 MHz region suggests a minor scalar contribution to the overall relaxivity. The relaxivity values are relatively low across the field range, consistent with the low spin multiplicity of Cu­(II) (*S* = 1/2) and in line with those reported for VO^2+^ chelates.^34^ The temperature dependence of *r*_1_ supports a fast-exchange regime for inner-sphere water molecules, as relaxivity decreases with increasing temperature in the high field region of the NMRD profiles. In this region, inner-sphere relaxivity is mainly affected by the rotational correlation time and the residence time of the coordinated water molecule(s), which have opposite temperature dependence. Increasing temperature shortens τ_R_, which results in a decrease of the observed relaxivity if water exchange is fast enough so that τ_M_ is not limiting the relaxation of the coordinated water molecule.^32^ To obtain a more accurate characterization of water exchange dynamics, VT ^17^O NMR measurements were performed (Figures 7B and 8C).

The ^1^H NMRD profiles recorded for [Cu­(TREN)]^2+^ at pH 12.0 display distinct features compared with those at neutral pH. In particular, a pronounced dispersion centered around 0.1–0.2 MHz is observed at low magnetic fields, indicating a significant scalar relaxation contribution in addition to the dipolar mechanism. A major difference is also evident in the temperature dependence of the ^17^O relaxivity, which reveals the absence of inner-sphere water molecules in exchange with the bulk at basic pH. Conversely, at neutral pH, inner-sphere water exchange is clearly detected. This behavior reflects the stronger interaction between OH^–^ and the metal center, which effectively suppresses ^17^O exchange under these conditions. The temperature-dependent ^1^H NMRD profiles and the ^17^O NMR data collected for the hydrated [Cu­(TACN)]^2+^ and [Cu­(TREN)]^2+^ complexes were simultaneously fitted using the well-established Solomon–Bloembergen–Morgan^28–30^ and Swift–Connick^94^ equations. For the [Cu­(TREN)]^2+^ complex at pH 12, only the ^1^H NMRD profiles were fitted, as the ^17^O NMR chemical shifts and relaxation rates showed negligible variations. In this analysis, a field-independent electronic relaxation time (τ_S_) for Cu­(II) was assumed.^95^ Given the large number of fitting parameters, several were fixed to known or literature values, while others were constrained based on EPR and computational studies. The fitting was performed assuming two inner-sphere water molecules for [Cu­(TACN)]^2+^ and a single coordinated water molecule for [Cu­(TREN)]^2+^. For the [Cu­(TREN)]^2+^ complex at pH 12, different fitting models were tested, indicating that the primary inner-sphere relaxation contribution arises from the hydroxide proton rather than the amine protons. Accordingly, the number of inner-sphere protons was fixed to one. The closest metal-proton distance for outer-sphere water molecules was fixed at 3.6 Å, a value estimated from classical MD simulations.^96^ Although such simulations have inherent limitations in accurately modeling the first coordination sphere of Cu­(II) complexes, they provide useful insight into the outer coordination shell and the rotational dynamics of the system (Figures S5 and S6).

The diffusion coefficient was fixed to 2.3 × 10^–9^ m^2^/s, and the associated activation energy was set to 20.0 kJ mol^–1^. The average distance between the water protons and the metal center was fixed to the values obtained from the combined ENDOR/DFT analysis: 2.56 Å for [Cu­(TACN)]^2+^ and 2.48 Å for [Cu­(TREN)]^2+^ (Table 2). For the [Cu­(TREN)]^2+^ complex at pH 12, the distance to the hydroxide proton was set to 2.40 Å, as determined by DFT calculations. The fits obtained for [Cu­(TACN)]^2+^ and [Cu­(TREN)]^2+^ accurately reproduce the experimental data, yielding electron relaxation times (τ_S_) of 0.98 and 0.54 ns, in agreement with values reported for other Cu­(II) systems.^97^ The value of τ_S_ increases significantly upon deprotonation of the coordinated water molecule in the complex of TREN (Table 3).

**Table 3:** Parameters Obtained from the Fitting of the ^1^H NMRD and ^17^O NMR Data for [Cu­(TACN)]^2+^ and [Cu­(TREN)]^2+^

	[Cu(TACN)]^2+^ (pH 4.5)	[Cu(TREN)]^2+^ (pH 7.4)	[Cu(TREN)]^2+^ (pH 12.0)
^298^ *r*_1_ (mM^–1^ s^–1^) (20 MHz)	0.31	0.23	0.20
^310^ *r*_1_ (mM^–1^ s^–1^) (20 MHz)	0.25	0.19	0.15
τ_S_ (ns)	0.98 ± 0.28	0.54 ± 0.07	1.13 ± 0.06
τ_R_ (ps)	10.6 ± 0.3	10 ± 1	10.4 ± 0.3
*E*_R_ (kJ mol^–1^)	20.2 ± 0.6	18.1 ± 1.1	17.9 ± 1.4
^298^τ_M(H_2_O)_ (ns)	0.075 ± 0.002	253 ± 15	–
^298^τ_M(H)_ (ns)	–	–	1.5 ± 0.2
Δ*H*_M_ (H_2_O) (kJ mol^–1^)	9.0 ± 0.3	40.0 ± 2.7	–
Δ*H*_M_ (H^+^) (kJ mol^–1^)	–	–	56.2 ± 4.3
Δ*S* ^⧧^ (J mol^–1^ K^–1^)	–20.9 ± 0.6	15.8 ± 0.9	74 ± 6
*A*_O_ **/** *ℏ* (10^8^ rad s^–1^)	2.82 ± 0.03 (44.9 ± 0.5 MHz)	2.0 ± 0.1 (31.8 ± 1.6 MHz)	–
*A*_H_/*ℏ* (10^6^ rad s^–1^)	8.3 ± 0.4 (1.3 ± 0.06 MHz)	3.8 ± 0.9 (0.61 ± 0.10 MHz)	21.04 ± 0.06 (3.39 ± 0.01 MHz)
*q*	2	1	–

The reorientational correlation times (τ_R_) are approximately 10 ps for all three systems investigated, in excellent agreement with the values obtained from EPR measurements (Table 1). The τ_R_ values were also estimated using classical MD simulations for the [Cu­(TACN)]^2+^ and [Cu­(TREN)]^2+^ complexes, following a methodology analogous to that used for Gd­(III) complexes-based on the autocorrelation function of the Cu–OH_2_ vector.^98^ These simulations yielded τ_R_ values of 6 and 17 ps for [Cu­(TACN)]^2+^ and [Cu­(TREN)]^2+^, respectively (Figures S7–S9) (compared with 7.6 and 13 ps from EPR studies; Table 1). The excellent agreement between the rotational correlation times obtained by EPR, NMRD, and molecular dynamics confirms the reliability of the fitted and calculated parameters. Moreover, the activation energies for τ_R_ (*E*_R_) are very close to those determined for small Gd­(III) complexes.^99^

The hyperfine coupling constant for the oxygen atoms of the coordinated water molecules in [Cu­(TACN)]^2+^ was found to be 2.82 × 10^8^ rad s^–1^, while an excellent fit of the ^1^H NMRD profiles was achieved considering a proton coupling constant of 8.3 × 10^6^ rad s^–1^ (1.3 MHz), comparable to what measured by Q-band ENDOR spectra (Table 1). DFT calculations yield *A*_O_/ℏ and *A*_H_/ℏ values in excellent agreement with the experiment (2.9 × 10^8^ rad s^–1^ and 6.9 × 10^6^ rad s^–1^). The *A*_O_/ℏ value obtained for [Cu­(TREN)]^2+^ at pH = 7.4 of 2.0 × 10^8^ rad s^–1^ is in excellent agreement with DFT (2.9 × 10^8^ rad s^–1^). However, DFT and ENDOR afford a *A*_H_/ℏ value of ∼ 21 × 10^6^ rad s^–1^ (3.5 MHz), while our fits afford a much smaller value of 3.8 × 10^6^ rad s^–1^ (0.6 MHz). Although we do not have a definitive explanation for this discrepancy, it is likely that dynamic effects play a significant role at the temperatures used in the NMRD experiments. Further investigations employing ab initio molecular dynamics simulations will be required to test this hypothesis. The large scalar contribution of the hydroxo-complex of TREN arises from a large *A*_H_/ℏ value of 21.0 × 10^6^ rad s^–1^, which is again somewhat overestimated by DFT (38.9 × 10^6^ rad s^–1^).

A very short water residence lifetime of 75 ps, associated with a low activation enthalpy of Δ*H*_M_ = 9.0 kJ mol^–1^, was obtained for [Cu­(TACN)]^2+^. The water exchange process is characterized by a negative activation entropy, Δ*S* ^‡^ = −20.9 ± 0.6 J mol^–1^ K^–1^.^100^ These parameters are similar to those reported for the aqua-ion [Cu­(H_2_O)_6_]^2+^: ^298^τ_M_(H_2_O) = 227 ps, Δ*H*_M_ = 11.5 kJ mol^–1^ and Δ*S* ^‡^ = −21.8 J mol^–1^ K^–1^. The very fast water exchange regime observed for [Cu­(H_2_O)_6_]^2+^ can be attributed to the strong dynamic Jahn–Teller effect that distorts the Cu­(II) coordination environment enhancing the lability of the coordinated water molecules.^82^ For [Cu­(TACN)]^2+^, the very fast exchange can be attributed to a low energy difference between the five-coordinated ground state and the six-coordinated transition state responsible for the associatively activated water exchange reaction, as suggested by the negative value of Δ*S* ^‡^. Conversely, ^298^τ_M_(H_2_O) is 3 orders of magnitude longer in [Cu­(TREN)]^2+^ compared to [Cu­(TACN)]^2+^. This is attributed to the trigonal bipyramidal geometry of the complex imposed by the tripodal ligand, which results in a rather compact structure. The sign of Δ*S* ^‡^ is positive in contrast to that obtained for [Cu­(TACN)]^2+^, but additional studies on other Cu­(II) complexes are needed to determine whether the sign of Δ*S* ^‡^ can provide mechanistic insight into water exchange processes. Nevertheless, the positive Δ*S* ^‡^ value and large activation enthalpy are consistent with a dissociatively activated mechanism.^101^

At basic pH, the best-fitting of the ^1^H NMRD profiles suggests the presence of a prototropic exchange process, catalyzed by the highly basic environment, involving the proton of a metal-bound hydroxide. Indeed, it has been shown that the coordinated water molecule in the Cu­(II) complex of TREN is characterized by a p*K*a of 9.4 to form the hydroxo complex [Cu­(TREN)­(OH)]^+^, which is the main species in solution at pH 12.^88,102^ This is in full agreement with our relaxometric and spectrophotometric data shown in Figure 6. Thus, the prototropic exchange must proceed by the base-catalyzed deprotonation of the bound hydroxide ligand, presumably forming a transient oxo-complex. Thus, the exchange rate (*k*) can be defined by the following equation:

$$\frac{1}{\tau_{M}}=\frac{1}{\tau_{M\left(H_{2}O\right)}}+\frac{1}{\tau_{M\left(H\right)}}\left[OH^{-}\right]$$ 4

where τ_M_(H_2_O) and τ_M_(H) indicate the residence lifetime of the inner sphere water and of the proton involved in the prototropic exchange. Since the τ_M_(H_2_O) contribution is absent, as suggested by the ^17^O NMR data, the exchange process in only controlled by the prototropic mechanism catalyzed by the basic environment. For [Cu­(TREN)]^2+^ at pH 12.0, the prototropic exchange occurs with a ^298^τ_M_ of 147 ns, affording a value of ^298^τ_M_(H) of 1.5 ± 0.2 ns. A high activation enthalpy of 56.2 ± 4.31 kJ mol^–1^ and a large positive activation entropy (Δ*S* ^‡^ = +74 ± 6 J mol^–1^ K^–1^) were obtained from the fit. This may be related to the large energy required to break the O–H bond of the hydroxide ligand.

## Conclusions

This work demonstrates how a combined experimental–computational strategy can be used to disentangle the structural, electronic, and dynamic factors governing relaxivity in Cu­(II)-based MRI probes. By integrating EPR spectroscopy (CW and pulsed ENDOR), variable-field and variable-temperature NMR relaxometry, and DFT/MD modeling, we provide a self-consistent description of both electronic structure and relaxation pathways in two prototypical Cu­(II) complexes, [Cu­(TACN)]^2+^ and [Cu­(TREN)]^2+^.

The results show that Cu­(II) relaxivity is not an intrinsic limitation of the *S* = 1/2 electronic configuration, but is instead dictated by a delicate interplay between coordination geometry, hydration state, and exchange dynamics. EPR and ENDOR experiments directly access key microscopic parameters, rotational correlation times, metal–proton distances, and hyperfine couplings, that are otherwise treated as adjustable quantities in relaxometric models. The excellent agreement between EPR-derived τ_R_ values, relaxometric fits, and molecular dynamics simulations validates the robustness of this integrated approach.

The pH-dependent studies reveal that Cu­(II) relaxivity is highly sensitive to protonation equilibria. This pH-dependent effect is not merely a complication but may represent a tunable parameter for designing responsive or environment-sensitive probes, providing that the relaxivity response to pH can be tuned to occur in the physiologically relevant pH range.

Overall, the results establish a coherent structure–relaxivity framework for Cu­(II) complexes and illustrate how the number of coordinated water molecules, exchange dynamics, and geometry dictate the balance between dipolar and scalar relaxation contributions. Furthermore, based on the analysis of the ^1^H NMRD profiles and the ^17^O NMR data, it was observed that the molecular and dynamic parameters that differ the most between the two compounds, due to their distinct geometry and molecular structure, are the hydration state of the metal center (*q* = 2 for [Cu­(TACN)]^2+^ and *q* = 1 for [Cu­(TREN)]^2+^), the electronic relaxation time (which is longer for [Cu­(TACN)]^2+^), and the mean residence time of the bound water molecules, which is significantly longer for [Cu­(TREN)]^2+^ (on the order of hundreds of ns). This is attributed to the trigonal bipyramidal geometry of the [Cu­(TREN)]^2+^ complex imposed by the tripodal ligand, which results in a rather compact structure.

The multitechnique approach used here allows for a very detailed understanding of the parameters that affect the relaxivity of Cu­(II) complexes. The methodology developed in this work will allow the accurate characterization of different families of Cu­(II) complexes, so that eventually contrast agent candidates can be designed on a rational basis. Indeed, the relaxivities observed for the complexes investigated in this work are rather low when compared with the Gd­(III) agents used in clinical practice, as well as the Cu­(II) protein system reported by Peacock.^21^ This work provided the tools that are required to understand the origin of these differences and therefore aid Gd­(III)-free contrast agent development.

From a design perspective, this study identifies concrete parameters that can be targeted to enhance Cu­(II)-based MRI contrast agents: control of coordination geometry to tune water-exchange regimes and electron relaxation time, modulation of protonation equilibria to activate scalar relaxation pathways, and optimization of hydration and rotational dynamics. For chelates with comparable metal hydration state, the residence time of bound water molecules exerts negligible influence on longitudinal relaxivity at clinical magnetic fields; in both [Cu­(TACN)]^2+^ and [Cu­(TREN)]^2+^ complexes, this parameter is sufficiently short to facilitate a fast exchange regime. A critical distinction arises, however, when comparing these to conventional low-molecular-weight Mn­(II) and Gd­(III) complexes. While the relaxivity of the latter at 1.5–3 T is primarily governed by the rotational correlation time (τ_R_), Cu­(II) and also Fe­(III) probes are significantly impacted by the electronic relaxation time (τ_S_). Therefore, in these cases, to optimize the *r*_1_ value, it is important to consider not only the molecular dimensions of the complex but also the molecular geometry that is strongly associated with τ_S_ value.

More broadly, the methodology presented here provides a transferable framework for rationally evaluating and designing first-row transition-metal MRI probes, bridging the gap between electronic structure, molecular dynamics, and macroscopic relaxometric performance.

## Supplementary Material



## Notes

Details on ENDOR experiments, MD simulations, and DFT data for [Cu­(TACN)]^2+^ and [Cu­(TREN)]^2+^ complexes (PDF)
