# Supplementary material for: Investigating Cu(II) Complexes for MRI: A Comprehensive Approach Using EPR, Relaxometry, and Computational Modeling
Source: Inorg Chem. 2026 Mar 3;65(10):5639–52. doi: 10.1021/acs.inorgchem.5c05926 (PMC12997156; doi:10.1021/acs.inorgchem.5c05926)
Supplement: Supplementary file 1 [file ic5c05926_si_001.pdf]

Supporting Information

Investigating Cu(II) Complexes for MRI: A Comprehensive Approach using EPR, Relaxometry, and Computational Modeling

Maria Chiara Pagliero,[a,§] *Marco Ricci,*[b,§] *Raúl Alvarado,*[c] *Carlos Platas-Iglesias,*[c] *Enrico Salvadori,*[a] *Valeria Lagostina,*[a] *Mario Chiesa,*[a*] *Mauro Botta,*[b,d] *Fabio Carniato*[b,d*]

[a] Department of Chemistry, University of Turin, Via Giuria 9, 10125 Torino, Italy. mario.chiesa@unito.it

[b] Dipartimento di Scienze e Innovazione Tecnologica, Università del Piemonte Orientale, Viale Teresa Michel 11, 15121 Alessandria, Italy. fabio.carniato@uniupo.it

[c] Centro Interdisciplinar de Química e Bioloxía (CICA) and Departamento de Química, Facultade de Ciencias, Universidade da Coruña, 15071 A Coruña, Galicia, Spain.

[d] Magnetic Resonance Platform (PRISMA-UPO), Università del Piemonte Orientale, Italy.

[§] These Authors contributed equally

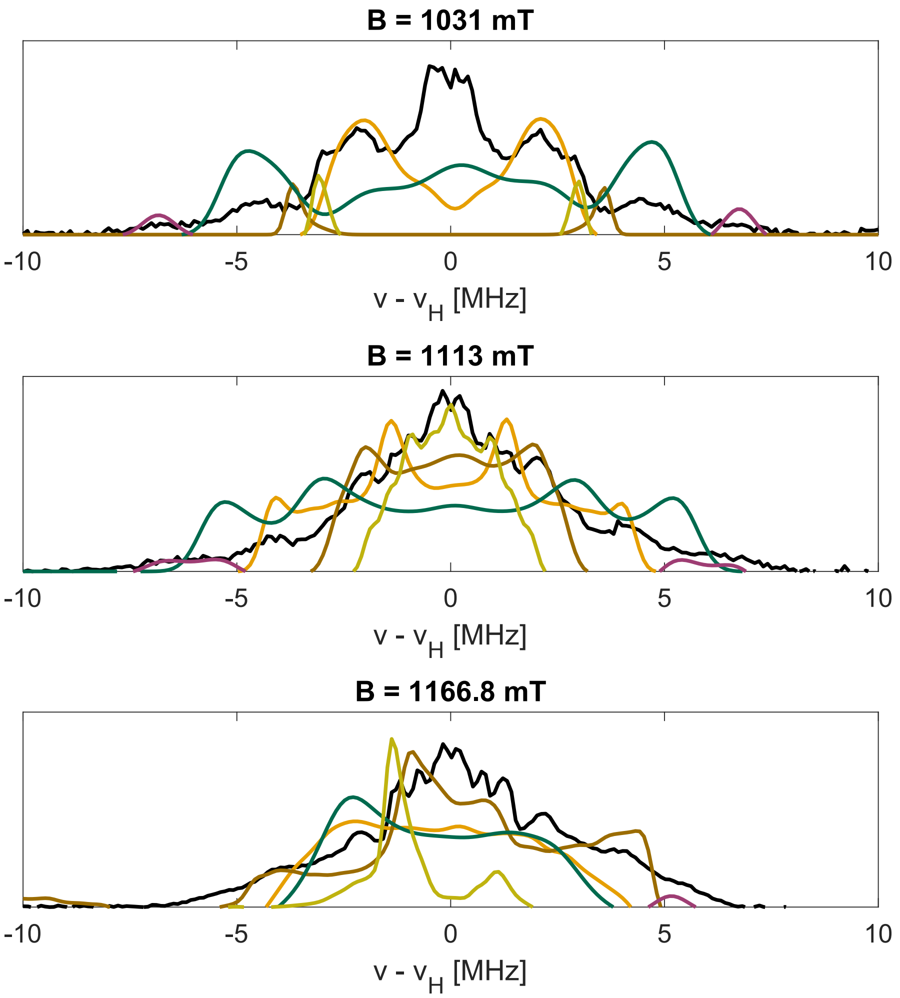

Figure S1. Deconvolution of Q-band ^{1}H Davies ENDOR for $[Cu(TACN)]^{2+}$ (black trace) at three different field positions. The individual contributions refer to the spin Hamiltonian parameters reported in Table S1 with the corresponding color code.

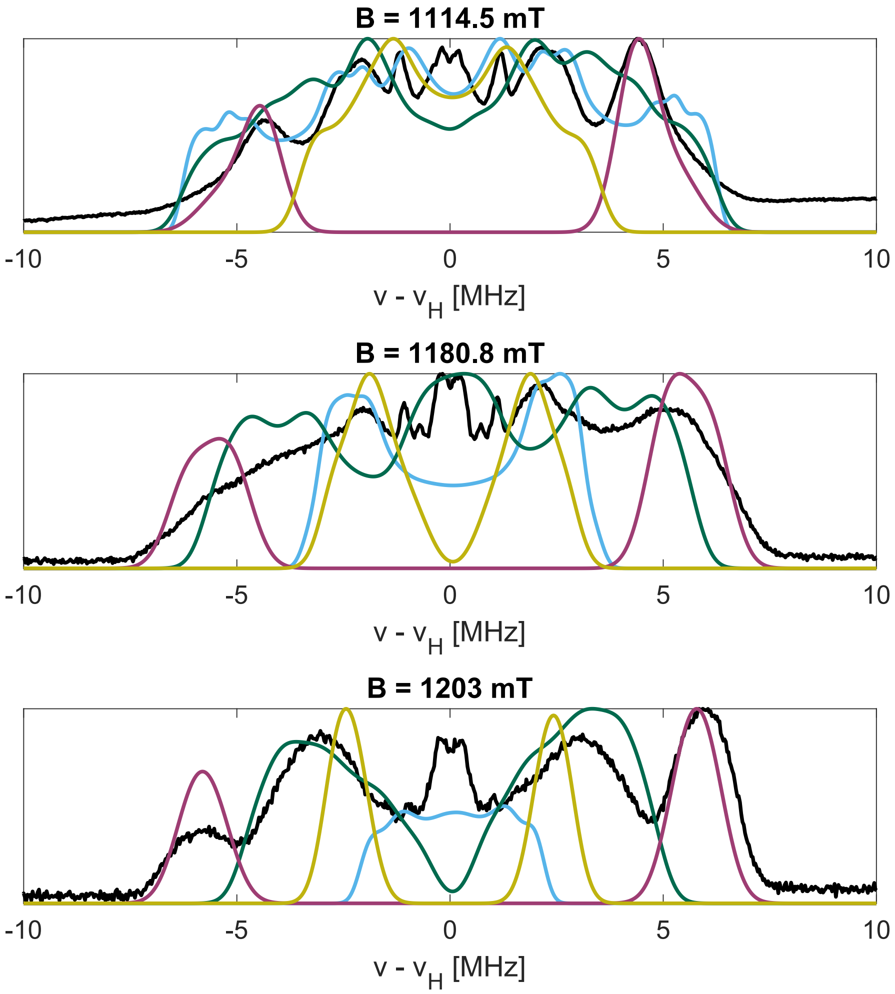

Figure S2. Deconvolution of Q-band ^{1}H Davies ENDOR for $[Cu(TREN)]^{2+}$ (black trace) at three different field positions. The individual contributions refer to the spin Hamiltonian parameters reported in Table S2 with the corresponding color code.

ELDOR detected NMR (EDNMR) experiments.

EDNMR experiments (Schosseler, P.; Wacker, T.; Schweiger, A. Pulsed Eldor Detected Nmr. *Chemical physics letters*, **1994**, 224(3-4), 319-324 b) Goldfarb, D. Hyperfine Spectroscopy–ELDOR-detected NMR. *EPR Spectroscopy: Fundamentals and Methods* **2018**, 359.) were performed using a Bruker SpinJet-AWG at 10K. Spectra were obtained with the pulse sequence:HTA $- T - \pi/2 - \tau - \pi - \tau -$echo with $t_{\mathrm{HTA}} = 9000$ ns, $T = 1000$ ns, $t_{\pi/2} = 400$ ns, $\tau = 600$ ns. A Gaussian shaped ELDOR pulse was used. The integration width of the echo was set to 800 ns, centered around the maximum of the spin echo. The central hole at $\Delta\nu = 0$ was removed in the EDNMR spectra by subtracting a fitted Lorentzian lineshape. Simulation of the

EDNMR spectra was performed using a simulation algorithm based on the work by Cox *et al.* (Cox, N.; Nalepa, A.; Lubitz, W.; Savitsky, A. ELDOR-detected NMR: A general and robust method for electron-nuclear hyperfine spectroscopy?. J. Magn. Reson. 2017, 280, 63–78.) within the Easyspin toolbox.(Stoll, S.; Schweiger, A. EasySpin, a comprehensive software package for spectral simulation and analysis in EPR. J. Magn. Reson. 2006, 178, 42–55.)

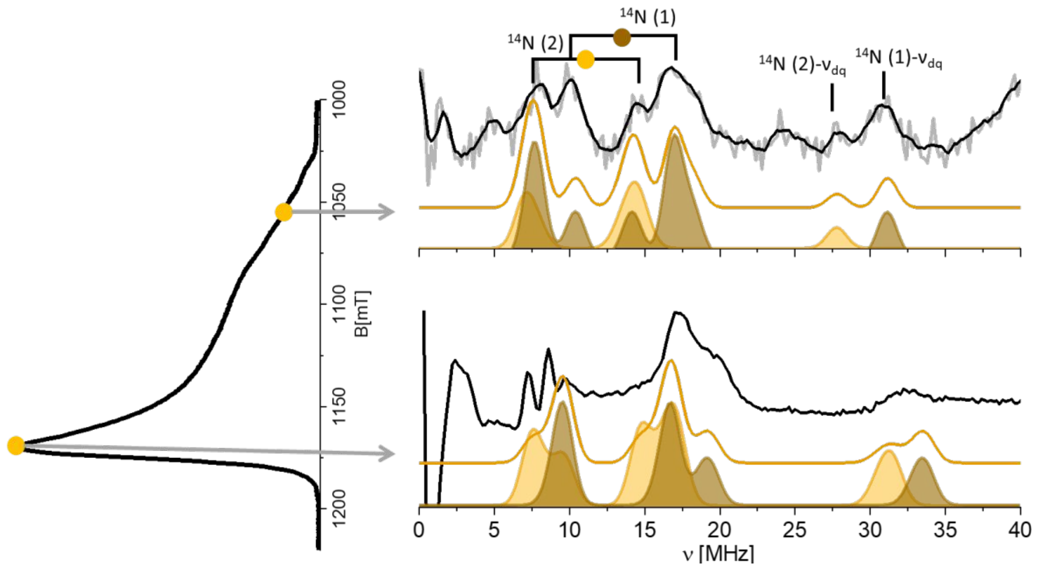

Figure S3. Q-band EDNMR spectra and corresponding computer simulations (shaded areas) for $[Cu(TACN)]^{2+}$. The magnetic field setting at which each spectrum was recorded are indicated by dots on the Echo detected EPR spectrum shown on the left panel. The hyperfine coupling values extracted from the simulation are listed in Table S1.

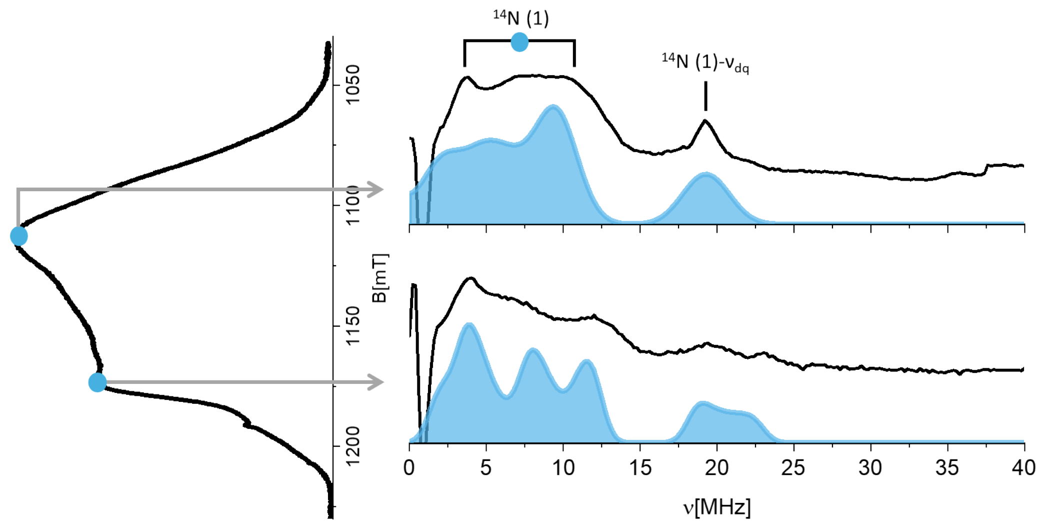

Figure S4. Q-band EDNMR spectra and corresponding computer simulations (shaded areas) for $[Cu(TREN)]^{2+}$. The magnetic field setting at which each spectrum was recorded are indicated by dots on the Echo detected EPR spectrum shown on the left panel. The hyperfine coupling values extracted from the simulation are listed in Table S1.

	g_x	g_y	g_z	${}^{Cu}A_x$ [MHz]	${}^{Cu}A_y$ [MHz]	${}^{Cu}A_z$ [MHz]	${}^{N}A_x$ [MHz]	${}^{N}A_y$ [MHz]	${}^{N}A_z$ [MHz]	α, β, γ [°]	${}^{N}Q$ [MHz]	η	α, β, γ [°]
**$Cu[(TACN)]^{2+}$**	2.058	2.058	2.288	30	30	-482	${}^{N(1)}$24	24	27	(0,-90,0)	2	-1.6	(0,30,0)
	-	-	-	-	-	-	${}^{N(2)}$20	21	27	(0,-90,0)	1.9	-1	(0,0,0)
**$[Cu(TREN)]^{2+}$**	2.191	2.207	2.005	-346	-332	180	${}^{N(1)}$9	9	17.5	(70,30,90)	3	-0.8	(0,75,-70)

Table S1. Spin-Hamiltonian parameters derived from the simulation of the CW-EPR spectra and EDNMR spectra.

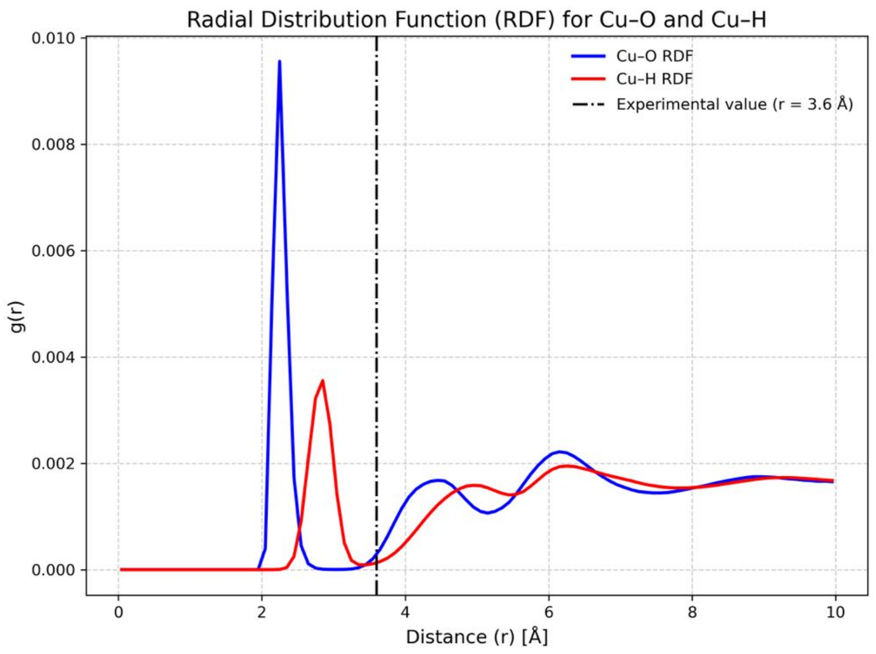

Figure S5. Cu-O and Cu···H Radial distribution functions (RDFs) involving water molecules, obtained from classical molecular dynamics simulations for the $[Cu(TACN)]^{2+}$ complex. The sharp peaks at 2.24 and 2.86 Å correspond to O and H atoms of inner-sphere water molecules. The vertical line at 3.6 Å corresponds to the estimate for the distance of closest approach of an outer-sphere water molecule.

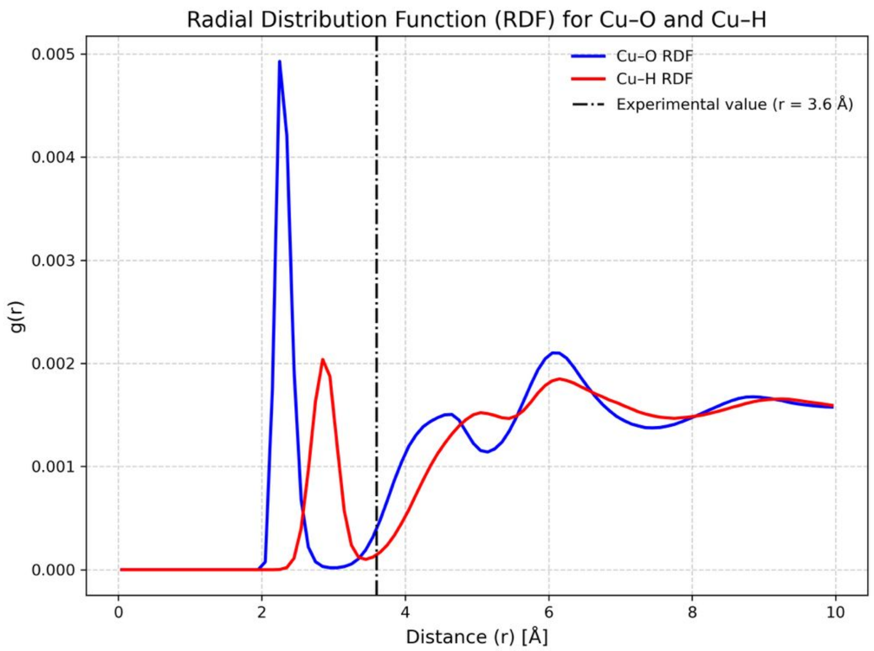

Figure S6. Cu-O and Cu···H Radial distribution functions (RDFs) involving water molecules, obtained from classical molecular dynamics simulations for the $[Cu(TREN)]^{2+}$ complex. The sharp peaks at 2.25 and 2.83 Å correspond to O and H atoms of the inner-sphere water molecule. The vertical line at 3.6 Å corresponds to the estimate for the distance of closest approach of an outer-sphere water molecule.

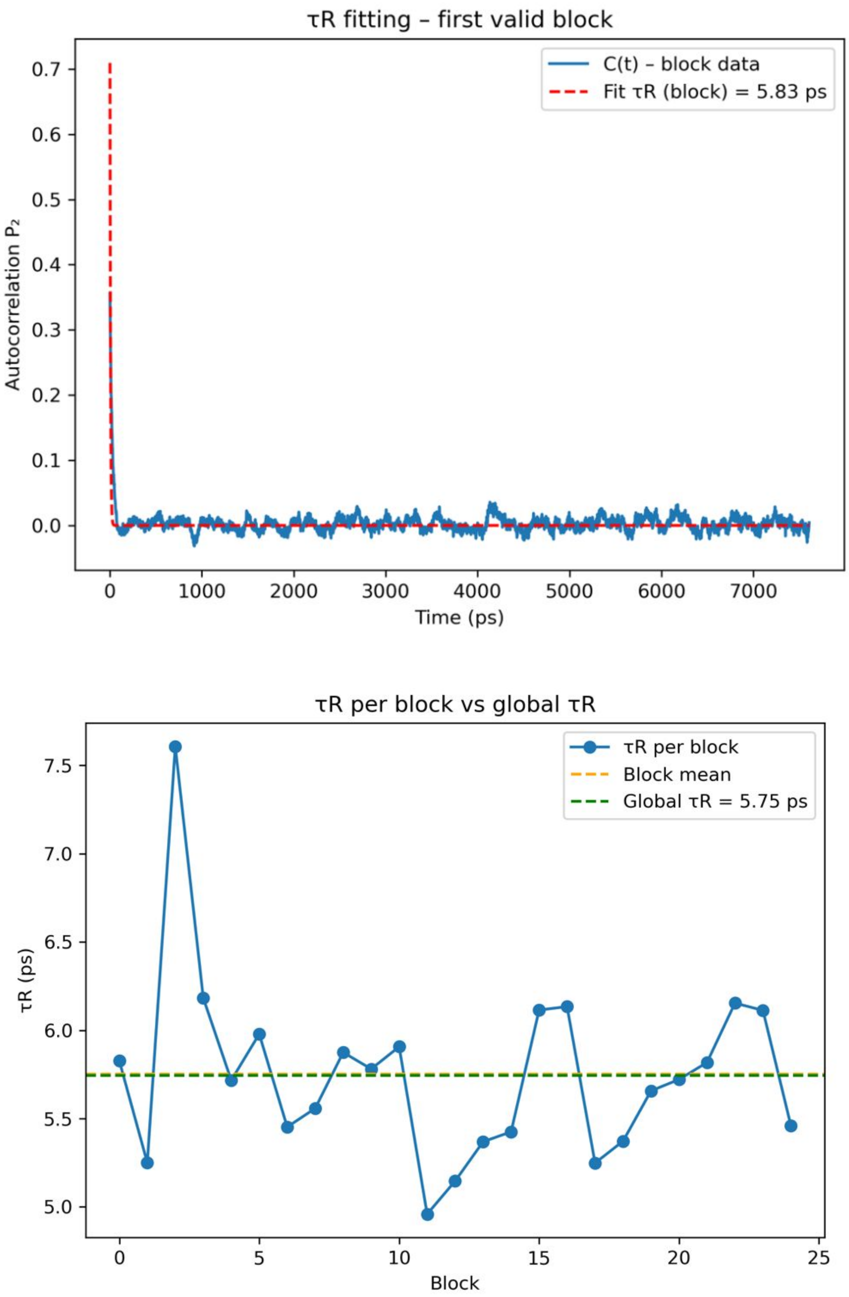

Figure S7. Top: Autocorrelation function used to estimate τ_R from classical MDs for $[Cu(TACN)]^{2+}$, affording τ_R = 5.8 ps. Bottom: The values of τ_R calculated using different blocks taking from equal steps of 2 ns along the MD trajectory.

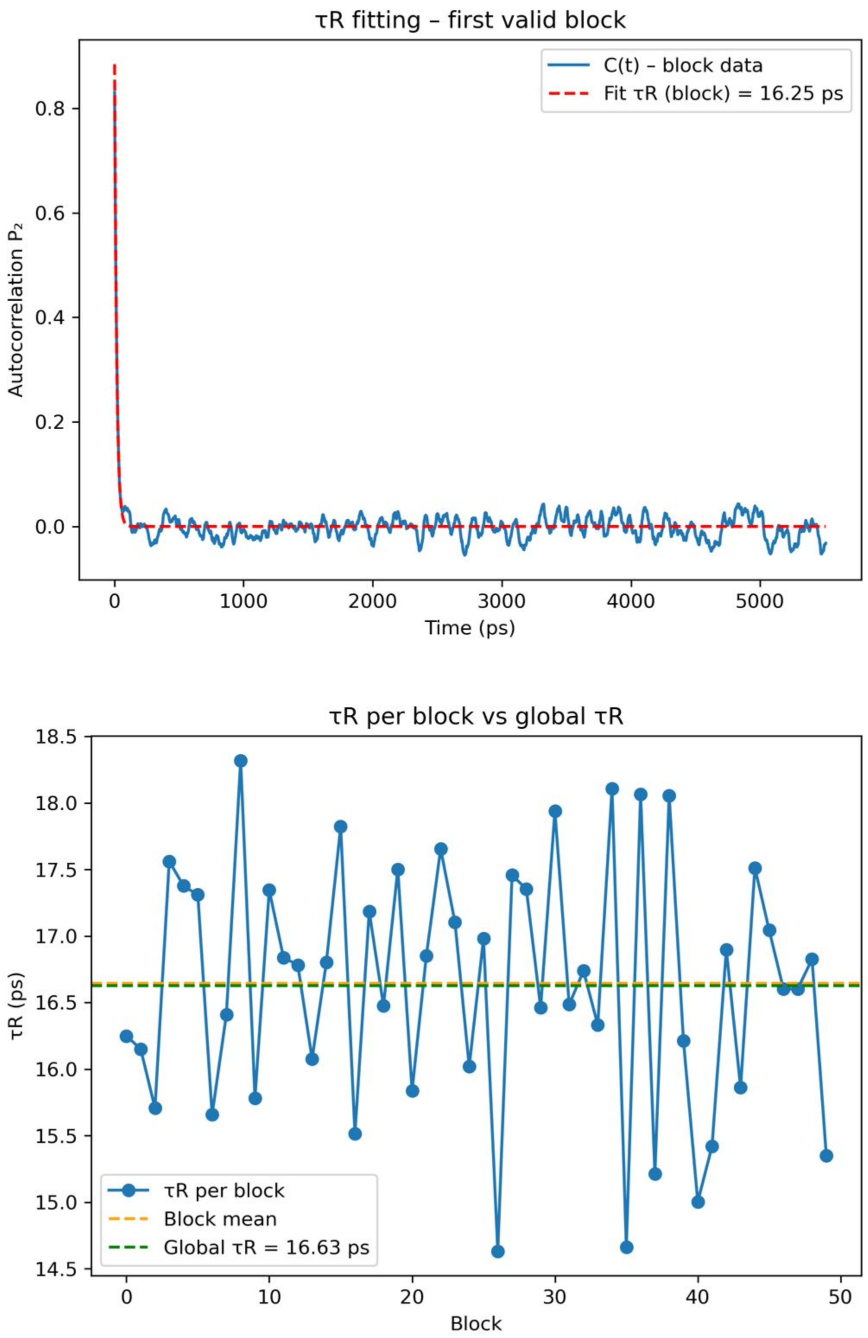

Figure S8. Top: Autocorrelation function used to estimate τ_R from classical MDs for $[Cu(TREN)]^{2+}$, affording τ_R = 16 ps. Bottom: The values of τ_R calculated using different blocks taking from equal steps of 2 ns along the MD trajectory.

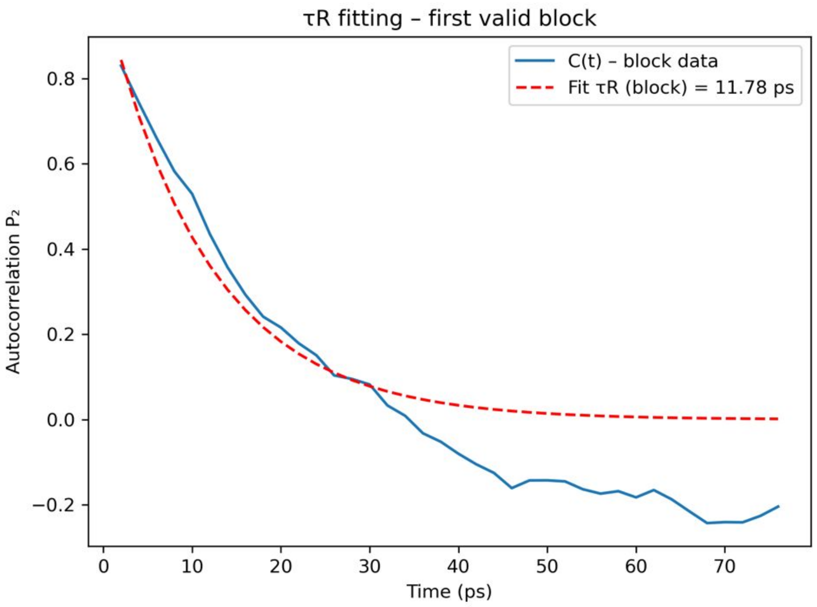

Figure S9. Autocorrelation function used to estimate τ_R from classical MDs for $[Cu(TREN)]^{2+}$ over a short period of 80 ps, affording τ_R = 12 ps.

Table S2. Simulated and DFT-computed ^{1}H hyperfine tensors (in MHz) arising from the coordinated water and the scaffold ligand in $[Cu(TACN)]^{2+}$. Relative orientations (α, β, γ) of the hyperfine tensors with respect to the g-frame are reported in degrees.

	A_x	A_y	A_z	α	β	γ
**27H (H_2O)**	-2.80±0.3	8.75±0.8	-8.5±0.8	47.3	47.3	-18.4
**DFT**	-2.89	7.47	-8.32			
**28H(H2O)**	9.5±1	-1.95±0.2	-7.5±0.5	-162	9.8	-154.5
**DFT**	9.02	-0.95	-7.23			
**2H (N)**	-5±0.4	6.85±0.8	-11.62±1.0	-100.4	27.6	142.9
**DFT**	-6.2	6.85	-16.62			
**4H (N)**	-2.9±0.3	-2.7±0.3	6.3±0.6	118.5	7.0	-134.8
**DFT**	-2.31	-2.47	5.64			
**12H (C)**	9.75±0.8	10.25±0.8	14.5±1.0	-53.5	25.2	15.0
**DFT**	19.58	20.21	24.70			

Table S3. Simulated and DFT-computed ^{1}H hyperfine tensors (in MHz) arising from the coordinated water and the scaffold ligand in $[Cu(TREN)]^{2+}$. Relative orientations (α, β, γ) of the hyperfine tensors with respect to the g-frame are reported in degrees.

	A_x	A_y	A_z	α	β	γ
**1H (H_2O)**	-5.2±0.3	-11.8±1.0	5.7±0.4	4.3	44.7	-1.3
**DFT**	-5.19	-12.3	6.69			
**11H (N)**	-6±0.3	4.5±0.3	-12±1.0	24.4	38.0	4.0
**DFT**	-5.94	4.49	-11.27			
**9H (N)**	-5.75±0.5	-2.8±0.3	7±0.8	-87.2	10.5	108.4
**DFT**	-5.75	-2.77	5.99			
**14H (C)**	12.8±1.0	9.0±0.8	8.2±0.8	-69	18	101
**DFT**	27	20	23.6			

Table S4. Cartesian coordinates (Å) obtained for the $[Cu(TREN)(H_2O)]^{2+}\cdot 7H_2O$ system with DFT calculations.

Cu	-4.197497	18.357257	2.565997
H	-2.841525	17.536457	4.446798
H	-2.337176	19.010820	4.092249
N	-5.259160	18.155437	0.823968
N	-5.318252	16.712333	3.218761
H	-4.757813	16.074149	3.780154
H	-6.095313	17.024037	3.796693
N	-5.197818	20.196043	2.761091
H	-5.542681	20.325334	3.715958
H	-4.570285	20.966951	2.547948
N	-2.521326	18.214724	1.361691
H	-1.807391	18.885944	1.635949
H	-2.128135	17.276206	1.485372
C	-6.317178	17.131285	1.047018
H	-6.631238	16.715363	0.086368
H	-7.175483	17.632638	1.496304
C	-5.813218	16.055054	1.991760
H	-6.613479	15.339083	2.196384
H	-4.976709	15.510688	1.552966
C	-5.835930	19.492634	0.519586
H	-6.645701	19.392582	-0.209302
H	-5.044588	20.101384	0.081761
C	-6.316968	20.158397	1.798053
H	-6.699803	21.155728	1.565279
H	-7.126812	19.588921	2.256294
C	-4.292121	17.718736	-0.219936
H	-4.710166	17.902502	-1.213689
H	-4.150634	16.643259	-0.105443
C	-2.962714	18.426991	-0.031310
H	-2.238484	18.042313	-0.754830
H	-3.067867	19.500021	-0.193991
O	-3.146775	18.473019	4.284212
O	-2.618188	15.866817	4.462323
H	-2.192867	15.612574	3.603168
H	-2.065377	15.493788	5.161441
O	-1.048215	19.994701	3.466412
H	-0.184109	19.812037	3.862375
H	-1.195310	20.941425	3.604559
O	-3.529502	21.844269	-0.826662
H	-4.005988	22.022639	-1.648937
H	-2.944253	22.606553	-0.720481
O	-1.618288	15.421567	1.992909
H	-2.091142	14.761921	1.424526
H	-0.677262	15.214837	1.913408
O	-2.988916	13.647218	0.445071
H	-2.403972	13.144115	-0.139739
H	-3.589431	14.115253	-0.152946
O	-6.288507	20.117500	5.561734
H	-5.769510	19.389180	5.931534
H	-7.182483	19.756459	5.479820
O	-7.276434	17.300195	-2.360289
H	-7.818355	18.007700	-2.735213
H	-7.446132	16.541762	-2.935436

Table S5. Cartesian coordinates (Å) obtained for the $[Cu(TACN)(H_2O)_2]^{2+}\cdot 7H_2O$ system with DFT calculations.

Cu	8.453587	2.993280	-4.009421
N	8.582871	5.152752	-4.475547
H	7.663674	5.592902	-4.462085
N	9.889894	3.459797	-2.637726
H	9.745434	2.847909	-1.834129
N	10.024853	2.839291	-5.296801
H	9.860110	2.139676	-6.025581
C	9.486871	5.757019	-3.478831
H	10.469375	5.911344	-3.927800
H	9.123962	6.737893	-3.162269
C	9.610853	4.861819	-2.243938
H	8.669932	4.849969	-1.692933
H	10.397901	5.239263	-1.583068
C	11.244631	3.240097	-3.211607
H	11.669262	4.207683	-3.477325
H	11.898876	2.782107	-2.468411
C	11.138294	2.355771	-4.443215
H	10.897568	1.330073	-4.158936
H	12.084304	2.352111	-4.994200
C	10.294796	4.152329	-5.948453
H	11.168476	4.598896	-5.473376
H	10.542612	3.989134	-6.998920
C	9.102049	5.104020	-5.848936
H	8.288413	4.749752	-6.484432
H	9.417859	6.088489	-6.216924
O	7.107142	2.861152	-2.497262
O	7.093816	2.361681	-5.351025
H	7.474680	2.303292	-1.765705
H	6.239127	2.487271	-2.769730
H	7.300914	1.456818	-5.738032
H	6.207090	2.274518	-4.935598
O	8.543716	1.587899	-0.624551
H	8.338604	1.754369	0.306557
H	8.640274	0.627863	-0.701014
O	4.832451	2.089655	-3.781513
H	4.103205	2.726077	-3.805138
H	4.423582	1.215725	-3.702693
O	7.706762	-0.008068	-6.339403
H	8.519758	0.193570	-6.869005
H	7.054789	-0.338992	-6.972037
O	12.860462	6.120520	-4.431805
H	13.494487	6.328555	-3.732092
H	13.111700	6.698375	-5.165108
O	5.626968	5.864328	-4.541925
H	5.316065	5.179886	-3.932881
H	5.350499	5.549147	-5.413732
O	9.959262	0.857883	-7.517651
H	10.798246	0.336121	-7.435154
H	9.914661	1.164447	-8.432978
O	12.268089	-0.488491	-7.102505
H	12.381445	-1.285102	-7.640399
H	13.030975	0.067153	-7.317394
